# Dynamic Chemical Profiling of *Lonicera japonica* Flos During the Maceration and Decoction Processes Integrating UPLC-MS and Molecular Networking

**DOI:** 10.3390/foods15081421

**Published:** 2026-04-19

**Authors:** Hui Ding, Chenglong Sun, Chuanzhi Kang, Yuemeng Liu, Xiao Wang, Lili Li

**Affiliations:** 1 Shandong Engineering Research Center for Innovation and Application of General Technology for Separation of Natural Products, Shandong Analysis and Test Center, Qilu University of Technology (Shandong Academy of Sciences), Jinan 250014, China; dinghui0925@126.com (H.D.); chenglongsun1989@163.com (C.S.); l19063439285@163.com (Y.L.); wangx@sdas.org (X.W.); 2 State Key Laboratory for Quality Ensurance, and Sustainable Use of Dao-di Herbs, National Resource Center for Chinese Materia Medica, China Academy of Chinese Medical Sciences, Beijing 100700, China; kangchuanzhi1103@163.com

**Keywords:** *Lonicera japonica* Flos, chemical profiling, molecular networking, maceration process, decoction process, UPLC-Q-TOF-MS, phenolic acids, flavonoids, iridoids

## Abstract

*Lonicera japonica* Flos (LJF) is widely used in pharmaceuticals and functional foods, with its bioactive constituents significantly influenced by processing methods. This study characterized the dynamic changes in chemical components in LJF under different maceration and decoction durations. Using UPLC-Q-TOF-MS and molecular networking, a total of 260 metabolites were unambiguously identified or tentatively characterized, including 66 iridoids, 42 flavonoids and 49 phenolic acids. Among these, 11 phenolic acids and 3 flavonoids were absent in the macerated samples. Twenty-two representative compounds were quantified using calibration curves. Most secondary metabolites, particularly phenolic acids, exhibited lower levels in the macerated samples than the decocted samples (e.g., 5-*O*-caffeoylquinic acid: 65.67–106.41 μg/g during maceration vs. 32,783.05–55,754.68 μg/g during decoction). The decoction process significantly enhances the extraction of active constituents. Notably, certain iridoids (e.g., 7-*O*-methyl morroniside: 92.91–354.59 μg/g during maceration vs. 50.43–171.40 μg/g during decoction) were better preserved under maceration, highlighting its advantage for retaining heat-sensitive bioactive components. During the decoction process, 5-hydroxycinnamoylquinic acids tended to transform into 3- and 4-hydroxycinnamoylquinic acid isomers. Most di-hydroxycinnamoylquinic acids and flavonoids significantly decreased after 30 min. Nitrogen-containing seco-iridoids declined rapidly after 15 min. To balance extraction efficiency with the preservation of heat-sensitive bioactive components, a decoction time of 15–30 min is recommended. The study systematically elucidates the dynamic changes in bioactive components under two preparation methods, offering critical insights and a scientific foundation for the precision utilization of LJF in pharmaceutical and functional food industries.

## 1. Introduction

*Lonicerae japonicae* Flos (LJF), the dried flower of *Lonicera japonica* Thunb., has been extensively utilized in the pharmaceutical and functional food industries [1,2,3]. Recognized for its broad-spectrum bioactivities, such as heat-clearing and detoxification, antioxidant, antitumor, anti-inflammatory, antiviral, antimicrobial and immunomodulatory effects [4,5,6], LJF serves as a critical component in over 500 traditional Chinese medicine formulations in China [7], such as Eunkyosan [8], Lianhuaqingwen capsules [9]. In recent years, LJF has attracted increasing attention as a promising raw material for food products. It has been incorporated into various functional foods, such as flavored alcoholic beverages [10], herbal teas with detoxifying functions [11], and fermented drinks exhibiting strong antioxidant activity [12]. Given the extensive applications of LJF in different industries, the efficient extraction of its bioactive compounds is essential for maximizing its functional benefits in supporting human nutrition and overall health.

The functional properties of LJF are closely associated with its characteristic phytochemical composition, mainly consisting of phenolic acids, flavonoids, and iridoids [13,14]. In both traditional medicinal practice and modern functional food applications, aqueous extraction—particularly maceration and decoction—represents the predominant method for preparing LJF infusions [15]. The processing parameters, especially temperature and duration, critically influence the extraction efficiency and structural stability of these bioactive constituents. The effects of post-harvest primary processing methods such as drying, steaming, and baking on the chemical composition of LJF have been extensively investigated [16,17]. These studies have demonstrated that thermal treatments significantly influence the contents of the chemical components. For the subsequent aqueous extraction process, which directly determines the composition of the final LJF infusion consumed by humans, existing studies have reported that heating duration affects the contents of several phenolic acids and flavonoids, thereby influencing antioxidant activities [18,19]. However, these investigations have primarily focused on a limited set of phenolic compounds (e.g., chlorogenic acid, luteoloside), while the dynamic changes in other important constituent classes, particularly iridoids, have received less attention. Notably, the value of such dynamic studies has been increasingly recognized. A metabolomics study of ginseng revealed that decoction time significantly influenced the transformation of ginsenosides, leading to increased levels of rare, more bioactive saponins [20]. In contrast, comparable systematic investigations for LJF are still lacking. Therefore, elucidating the dynamic changes in the chemical composition of LJF during these two preparation processes is important for guiding its proper use in both medicinal and functional food applications.

Metabolomics, which focuses on the global profiling of small-molecule metabolites, aligns well with the holistic philosophy of traditional Chinese medicine and has become a powerful tool in herbal medicine research [21,22]. Ultra performance liquid chromatography-mass spectrometry (UPLC-MS) is a dominant platform for comprehensive metabolomic analysis due to its high sensitivity, superior separation efficiency, and high-throughput capability [23,24]. However, a major bottleneck in UPLC-MS-based untargeted analysis of plant extracts like LJF is the reliable identification of metabolites, largely due to the limited coverage of specialized secondary metabolite databases [25,26]. Molecular networking has emerged as a powerful computational strategy to address this challenge. Organizing MS/MS data by spectral similarity enables the untargeted annotation of structurally related compounds and can help uncover novel chemical scaffolds in complex mixtures [27]. This approach is particularly well-suited for complex herbal matrices like LJF, where numerous structurally related metabolites coexist and can be efficiently annotated through MS/MS spectral similarity. In the present study, molecular networking was employed to facilitate the annotation of structurally diverse metabolites in LJF extracts across different processing conditions.

In this study, a UPLC-Q-TOF-MS-based metabolomics approach was employed to comprehensively analyze the chemical profiles of LJF subjected to different durations of maceration and decoction. The study focuses on characterizing the time-dependent changes in phytochemical composition during these two preparation processes, comparing the differential effects of maceration and decoction on the extraction and transformation of key bioactive constituents, and identifying optimal processing time windows to preserve specific classes of compounds. Chemical components were structurally annotated using MS/MS analysis and molecular networking, with further confirmation by available reference standards. In addition, 22 representative compounds were quantified using calibration curves with authentic standards. By systematically evaluating the time-dependent effects of these preparation methods, this study seeks to provide a scientific basis for the rational preparation of LJF in functional foods and herbal infusions.

## 2. Materials and Methods

### 2.1. Chemicals and Reagents

Acetonitrile and methanol (HPLC grade) were obtained from Merck (Darmstadt, Germany). Formic acid was purchased from Honeywell (Fluka, Germany). Ultrapure water was prepared using a Direct-Q 8 UV-R water purification system (Millipore, Billerica, MA, USA). Reference standards (purity) were purchased from Chengdu Desite Biological Technology Co., Ltd. (Chengdu, China), Shanghai Yuanye Bio-Technology Co., Ltd. (Shanghai, China), Jiangsu Yongjian Biological Technology Co., Ltd. (Taizhou, China), and Sichuan Weikeqi Biological Technology Co., Ltd. (Chengdu, China).

### 2.2. Sample and Reference Standard Solutions Preparation

The dried LJF materials were obtained from the Pingyi growing area in Shandong Province. The materials were identified as *Lonicera japonica* Flos by Professor Xiao Wang from Shandong Analysis and Test Center. The dried LJF samples were ground into powder and sieved through a 40-mesh screen. Accurately weighed portions (1.0000 ± 0.0002 g) of the powder were used for extraction. For maceration, a solid-to-liquid ratio of 1:50 was adopted with reference to previous reports on LJF [28]. In the maceration experiment, the powder was transferred into 100 mL conical flasks, mixed with 50 mL of deionized water, and macerated at room temperature (20 ± 2 °C) for 1 h, 2 h, 3 h, 6 h, 12 h, and 24 h. These time points were selected to capture the dynamic changes in chemical composition from initial release to potential degradation or transformation, reflecting common practices in both traditional herbal preparation and modern functional beverage applications. During decoction, to prevent the mixture from boiling dry during the extended heating period [20], the powder was placed in a casserole with 300 mL of deionized water, brought to a boil, and then simmered for 0, 15, 30 min, 1, 2, and 3 h. The 0 min time point was defined as immediately after boiling, serving as the baseline for decoction onset. The selected time points were chosen to capture the dynamic changes in chemical composition from the initial extraction to subsequent thermal degradation or transformation, reflecting common practices in both traditional Chinese medicine decoction and modern hot-water extraction for functional beverages. Although the initial water volumes differed between maceration and decoction, this difference was due to the practical requirements of the two procedures.

After extraction, all mixtures were centrifuged, and the supernatants were collected. The supernatants were freeze-dried using an EPSILON 2-4 freeze-drier (Martin Christ, Osterode am Harz, Germany) and then reconstituted in 10 mL of deionized water, normalizing the sample concentration based on the original dry weight (1 g) to ensure comparability across all time points, followed by a 10-fold dilution with methanol–water (1:1, *v*/*v*) prior to UPLC-MS analysis. The experiments were conducted with three independent replicates per time point. Quality control (QC) samples were prepared by mixing equal aliquots of all individual samples. The QC sample was injected at regular intervals throughout the analytical sequence to monitor system stability and analytical reproducibility.

A total of 22 reference standards, including 3-*O*-*p*-coumaroylquinic acid, 5-*O*-*p*-coumaroylquinic acid, 3-*O*-caffeoylquinic acid, 5-*O*-caffeoylquinic acid, 4-*O*-caffeoylquinic acid, 3-*O*-feruloylquinic acid, 5-*O*-feruloylquinic acid, 3,4-*O*-di-caffeoylquinic acid, 3,5-*O*-di-caffeoylquinic acid, 4,5-*O*-di-caffeoylquinic acid, luteoloside, isoquercitrin, kaempferol-3-*O*-glucoside, loganic acid, secologanoside, morroniside, epi-vogeloside, loganin, (E)-aldosecologanin, (Z)-aldosecologanin, sweroside and 7-*O*-methyl morroniside, were accurately weighed and individually dissolved in methanol to prepare stock solutions at a concentration of 1 mg/mL. All stock solutions were stored at 4 °C before use. Mixed standard working solutions at appropriate concentrations were prepared by serial dilution of the stock solutions with 50% (*v*/*v*) methanol in water prior to analysis. Detailed information on these reference standards (including suppliers and purity) is provided in the Supplementary Materials (Table S1).

### 2.3. UPLC-Q-TOF-MS Analysis for Untargeted Metabolomics and Targeted Quantification

All macerated and decocted samples were analyzed using an ultra-performance liquid chromatography system (ACQUITY H-class, Waters, Milford, MA, USA) coupled to a quadrupole time-of-flight (Q-TOF) mass spectrometer (Impact II, Bruker, Germany). Separation was achieved on an Agilent SB-C18 column (2.1 × 100 mm, 1.8 μm) maintained at 40 °C. The mobile phase consisted of (A) water with 0.1% formic acid and (B) acetonitrile. A gradient elution program was applied at a flow rate of 0.3 mL/min: 0–8 min, 5–10% B; to 18 min, 25% B; to 25 min, 100% B and kept for 5 min; to 30.1 min, 5% B, and kept for the end. The total elution time was 35 min. Prior to UPLC analysis, all samples were filtered through a 0.22 μm membrane filter (Jinteng, Tianjin, China) to remove particulate matter. The injection volume was 2 μL.

Mass spectrometry detection was performed in both positive and negative ionization modes with a mass scan range of *m*/*z* 50–1500. The capillary voltage was set to 3500 V (positive) and 3000 V (negative). The end plate offset was set to 500 V. The nebulizer pressure was 2.0 bar. The dry gas flow rate was 8.0 L/min. The drying gas temperature was 220 °C. The collision radio frequency (RF) was 750 Vpp. The prepulse storage was 6 μs, and the transfer time was 60 μs. Full-scan MS data were acquired for MS1 analysis. MS2 spectra were obtained in data-dependent acquisition (DDA) mode with a mass-dependent collision energy ramp ranging from 20 to 70 eV, increasing with precursor *m*/*z*. Mass calibration was performed using a sodium formate solution as the external calibrant before each analytical sequence. During sample analysis, continuous internal mass calibration was achieved using the Bruker instrument’s lock mass function, with sodium formate clusters introduced as reference ions via a syringe pump. This approach ensures high mass accuracy across the entire analytical run. The mass accuracy tolerance for metabolite identification was set to ≤5 ppm.

Meanwhile, 22 representative bioactive compounds were selected for quantitative analysis. Calibration curves were established using authentic standards, and the concentrations were determined by interpolating the peak areas onto the corresponding calibration curves (Table S2).

### 2.4. Method Validation

The analytical method was validated in accordance with the recommended practices for untargeted metabolomics studies [29]. Repeatability, intra-day precision, inter-day precision, and stability were evaluated using QC samples. Repeatability was assessed by analyzing six replicate QC samples. Intra-day precision was evaluated by analyzing QC samples six times within a single day. Inter-day precision was assessed by analyzing QC samples on three consecutive days. Stability was examined by analyzing QC samples at 6 h intervals over 48 h. For each validation parameter, the relative standard deviation (RSD) of peak areas was calculated as the primary acceptance criterion. For the targeted quantification of 22 compounds, linearity was evaluated using calibration curves constructed with more than five concentration levels (R^2^ > 0.99). The detailed validation procedure and results are provided in the Supplementary Materials (Table S2 and Figure S1).

### 2.5. Data Processing

Data deconvolution and peak alignment were performed using MS-DIAL (version 4.24) [30], yielding a peak table containing *m*/*z*, retention time, and peak area. Data processing was performed using MS-DIAL with a mass accuracy tolerance of 0.02 Da for MS1 and 0.05 Da for MS2. The minimum peak height was set to 1000, and the mass slice width was set to 0.1 Da. For peak alignment, the retention time tolerance was set to 0.1 min and the MS1 tolerance to 0.03 Da, with gap filling enabled to ensure comprehensive feature extraction across samples. Metabolite annotation was conducted based on accurate mass (mass error ≤ 5 ppm), MS/MS fragmentation patterns, and comparison with spectral databases, including GNPS, MassBank, and MS-DIAL public MS/MS libraries. Reference standards were used for further confirmation when available. Peaks with RSD > 30% in QC samples were excluded from further analysis. Principal component analysis (PCA) was conducted using MetaboAnalyst 6.0 [31]. Prior to PCA, the data were normalized by sum and log-transformed (base 10). Hierarchical cluster analysis (HCA) and one-way analysis of variance (ANOVA) were carried out using MultiExperiment Viewer (version 4.9.0). For HCA, the distance metric was set as Pearson correlation. The linkage method selection was set as average linkage clustering. For ANOVA, the *p*-values obtained from one-way ANOVA were further adjusted using the Benjamini–Hochberg false discovery rate (FDR). Fold change (FC) was then calculated separately for the decoction and maceration datasets, based on all pairwise comparisons between time points, using the ratio of mean peak areas between groups. In the decoction dataset, FC values were calculated for 0 min vs. 15 min, 30 min, 1 h, 2 h, and 3 h; 15 min vs. 30 min, 1 h, 2 h, and 3 h; 30 min vs. 1 h, 2 h, and 3 h; 1 h vs. 2 h and 3 h; and 2 h vs. 3 h. In the maceration dataset, FC values were calculated for 1 h vs. 2, 3, 6, 12, and 24 h; 2 h vs. 3, 6, 12, and 24 h; 3 h vs. 6, 12, and 24 h; 6 h vs. 12 and 24 h; and 12 h vs. 24 h. Thus, FC values were obtained for all pairwise time-point comparisons within each preparation method. Differential metabolites were screened using the criteria of *p*(FDR) < 0.05 and FC > 2. Feature-Based Molecular Networking was performed on the Global Natural Products Social Molecular Networking platform (GNPS) [32]. Min pairs cos was set as 0.7. The minimum number of matched fragment ions was set to 6. The precursor ion mass tolerance is 0.02 Da, and the fragment ion mass tolerance is 0.05 Da. All other parameters were maintained at the platform default values.

## 3. Results and Discussion

### 3.1. Metabolites Identification with Molecular Networking

**Figure 1:**
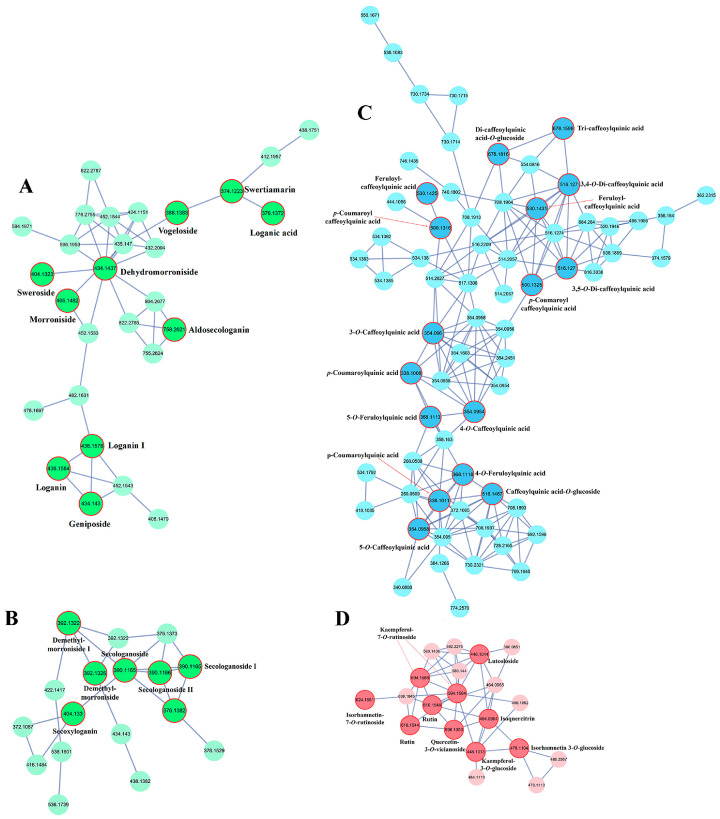
GNPS-based molecular networking analysis of LJF metabolites. Green clusters correspond to iridoids (**A**,**B**), blue clusters to phenolic acids (**C**), and pink clusters to flavonoids (**D**). Darker nodes denote the identified metabolites.

As a powerful computational and visualization tool, molecular networking has emerged as an efficient strategy for large-scale metabolite annotation and discovery. Organizing MS/MS data by spectral similarity enables rapid identification of both known and unknown metabolites. In the LJF molecular networking, a total of 850 mass spectral nodes were included (Figure S2), comprising 101 clusters (with nodes ≥ 2) and 331 single nodes. As shown in Figure 1, four major clusters were identified, corresponding to iridoids (clusters A and B), phenolic acids (cluster C), and flavonoids (cluster D). The annotation of each cluster was performed using a seed-based strategy. Briefly, compounds with authentic reference standards served as anchor nodes (e.g., loganic acid for Cluster A, secologanoside for Cluster B, 3-*O*-caffeoylquinic acid for Cluster C, luteoloside for Cluster D). Structurally related compounds within the same cluster were then annotated based on shared MS/MS fragmentation patterns, diagnostic fragment ions, and spectral matching against databases.

Using molecular networking and database matching, a total of 260 metabolites were unambiguously identified or tentatively characterized (Tables S3 and S4), including 66 iridoids, 42 flavonoids, 49 phenolic acids, 42 fatty acids, 16 amino acids, 13 organic acids, 10 nucleotides, 5 lipids, 5 dipeptides, 3 sugars, 2 vitamins, and 7 others. Following the Metabolomics Standards Initiative (MSI) framework [33], these metabolites were classified into two confidence levels: Level 1 (confirmed structure), assigned to compounds unambiguously identified by comparison with authentic reference standards; Level 2 was assigned to putatively annotated compounds and was further divided into three sublevels according to the annotation strategy used: Level 2^a^, compounds annotated based on database and/or literature comparison combined with MS/MS data; Level 2^b^, compounds annotated with support from molecular networking; and Level 2^c^, compounds annotated based on characteristic fragmentation pattern analysis. Among the 260 metabolites, 73 compounds were assigned as Level 1 based on available reference standards, while the remaining 187 were assigned as Level 2. Detailed information, including retention times, molecular formulas, MS/MS fragments, and identification confidence levels, is provided in Tables S3 and S4. Among the samples, 260 and 246 metabolites were detected in the decoction- and maceration-processed samples, respectively. Notably, 3 flavonoids and 11 phenolic acids were not detected in the macerated samples.

#### 3.1.1. Iridoids

In the molecular network, iridoids were primarily grouped into clusters A and B. Their fragmentation patterns were analyzed to annotate structurally related analogs. In cluster A (Figure 1A), loganic acid (*m*/*z*, 375.1297, mass error: 2.93 ppm), confirmed by a reference standard, served as the anchor node, and its detailed fragmentation pattern is shown in Figure S3A. It was connected to an unknown compound (*m*/*z*, 373.1139). Its MS/MS spectrum exhibited characteristic fragment ions at *m*/*z* 213 ([M-H-Glc]^−^), 169 ([M-H-Glc-CO_2_]^−^), 151 ([M-H-Glc-CO_2_-H_2_O]^−^) and 125 (RDA cleavage). These product ions correspond to the successive loss of a glucose (Glc) moiety, a carboxylic acid group (CO_2_), and a water molecule (H_2_O) from the aglycone. The neighboring compound (*m*/*z*, 373.1139) displayed analogous fragment ions at *m*/*z* 211, 167, 149, and 123, indicating the neutral loss of Glc, CO_2_, H_2_O, and RDA cleavage, respectively. Based on this fragmentation pattern and comparison with literature data [14], it was annotated as swertiamarin (*m*/*z*, 373.1139, mass error: 2.68 ppm). Similarly, Morroniside (*m*/*z* 405.1403, mass error: 2.96 ppm), identified using an authentic standard, served as the anchor node. It showed fragment ions at *m*/*z* 373, 243, 155, and 141, corresponding to neutral losses of methanol, Glc, and RDA cleavage [34]. The neighboring compound (*m*/*z*, 433.1361) showed fragment ions at 387, 225, 207, 155, and 123, indicating neutral losses of Glc and H_2_O, and RDA cleavage, respectively. Compared with morroniside, the neighboring compound (*m*/*z* 433.1361) was observed in the form of a formate adduct ([M+HCOO]^−^) and showed a molecular weight 18 Da lower. In comparison with reference [14], it was putatively identified as dehydromorroniside (*m*/*z*, 433.1361, mass error: 4.62 ppm). The compound (*m*/*z*, 403.1252) is connected with dehydromorroniside. It showed fragment ions at 357, 195, and 125, indicating the neutral loss of Glc and RDA cleavage, respectively. It was identified as sweroside (*m*/*z* 403.1252, mass error 4.47 ppm) and confirmed using standards. In cluster B (Figure 1B), secologanoside (*m*/*z*, 389.1087, mass error: 2.31 ppm), confirmed by a standard, served as the anchor node. It was connected to two unknown compounds (I and II). Secologanoside yielded fragment ions at *m*/*z* 345 ([M-H-CO_2_]^−^), 209 ([M-H-Glc]^−^), 183 ([M-H-Glc-C_2_H_2_]^−^) and 165 ([M-H-Glc-CO_2_]^−^). Neighboring compound I (*m*/*z*, 375.1294) produced fragment ions at *m*/*z* 195, 169, and 151, suggesting neutral losses of Glc, C_2_H_2_, and H_2_O, respectively. It was annotated as demethylsecologanol (*m*/*z* 375.1294; mass error 2.47 ppm). Neighboring compound II (*m*/*z*, 391.1242) showed fragment ions at *m*/*z* 229, 211, 193, and 123, indicating neutral losses of Glc, H_2_O, and RDA cleavage, respectively. This compound was consequently annotated as demethyl-morroniside (*m*/*z*, 391.1242, mass error: 2.05 ppm) [14]. Through molecular networking, a total of 24 iridoids were annotated. The analysis revealed common fragmentation pathways for this class, including neutral loss of Glc, H_2_O, C_2_H_2_, CH_3_OH, and carboxylic acid groups, as well as RDA cleavage. However, some iridoids were not captured in the molecular networks, likely because low-abundance compounds were not triggered for fragmentation in data-dependent acquisition (DDA) mode, or because suboptimal collision energy resulted in poor-quality MS/MS spectra. To address this, we performed targeted MS/MS analysis with optimized collision energies, which led to the further identification of 42 additional iridoids. As the major secondary metabolites in LJF, the 66 identified iridoids can be classified into three categories: 23 general iridoids, 26 seco-iridoids, and 16 nitrogen-containing seco-iridoids. All of these were detected in both the decocted and macerated samples.

#### 3.1.2. Phenolic Acids

Phenolic acids in LJF are commonly found conjugated with quinic acid or sugars. In cluster C (Figure 1C), 3-*O*-Caffeoylquinic acid, 4-*O*-caffeoylquinic acid, 4-*O*-*p*-coumaroylquinic acid and 5-*O*-feruloylquinic acid were grouped together and confirmed using reference standards. These four compounds served as anchor nodes for cluster C. Furthermore, di-caffeoylquinic acid (*m*/*z*, 515.1196, mass error: 2.33 ppm) displayed fragment ions at *m*/*z* 353, 191, 179, and 173, indicative of a mono-caffeoylquinic acid moiety, quinic acid, and caffeic acid. The detailed fragmentation pattern of the representative isomer 3,5-*O*-di-caffeoylquinic acid is shown in Figure S3B. Their MS/MS spectra exhibited characteristic fragment ions of the phenolic acid moieties (*m*/*z* 179 for caffeic acid, 163 for coumaric acid, 193 for ferulic acid) and quinic acid (*m*/*z* 191, 173). Positional isomers of these compounds were subsequently identified and similarly confirmed with standards. An unknown compound (*m*/*z*, 515.1404, mass error: 1.75 ppm) was also linked with this cluster. It produced fragment ions at *m*/*z* 353, 191, 179, and 173, corresponding to neutral losses of Glc, quinic acid, and caffeic acid, respectively. Then it was annotated as caffeoylquinic acid-*O*-glucoside. A neighboring compound (*m*/*z*, 529.1354, mass error: 2.65 ppm) yielded fragment ions at *m*/*z* 367, 353, 191, 179, and 173, which were characteristic of feruloylquinic acid, caffeoylquinic acid, quinic acid, and caffeic acid, respectively. Then it was annotated as feruloylcaffeoylquinic acid. By applying these fragmentation rules, a total of 49 phenolic acids were characterized. Notably, 11 phenolic acids were not detected in the macerated samples, including three feruloylcaffeoylquinic acid isomers, five *p*-coumaroyl caffeoylquinic acid isomers, two dicaffeoylquinic acid-*O*-glucoside isomers and tri-caffeoylquinic acid.

#### 3.1.3. Flavonoids

In cluster D (Figure 1D), luteoloside, kaempferol-7-*O*-rutinoside, and isoquercitrin were interconnected, and their structures were confirmed using reference standards. These three compounds served as anchor nodes for cluster D. Their MS/MS spectra exhibited neutral losses of sugar moieties along with characteristic fragment ions of the aglycones. For instance, luteoloside (*m*/*z*, 447.0936, mass error: 3.36 ppm) produced fragment ions at *m*/*z* 285 ([M-H-Glc]^−^), 217, 175, 151, and 133. Fragment ions at *m*/*z* 217, 175, 151, and 133 were characteristic of luteolin, and its detailed fragmentation pattern is shown in Figure S3C. By applying these fragmentation rules, various glycosylated derivatives of flavone and flavonol aglycones were identified. The characteristic aglycone ions were verified using reference standards. Additionally, acetylated and malonylated flavonoids were identified based on neutral losses of 42 Da (acetyl group) and 86 Da (malonyl group), respectively. Using this fragmentation-based approach, a total of 42 flavonoids were characterized. Among these, 42 and 39 flavonoids were detected in decocted and macerated samples, respectively. Specifically, quercetin-*O*-acetylglucoside, quercetin-*O*-malonylglucoside and eriodictyol-7-*O*-glucoside were not detected in the macerated samples. These well-characterized chemical constituents provide a foundational basis for further investigation into the effects of decoction and maceration time on the metabolite profile of LJF.

### 3.2. PCA in Macerated and Decocted LJF Samples

**Figure 2:**
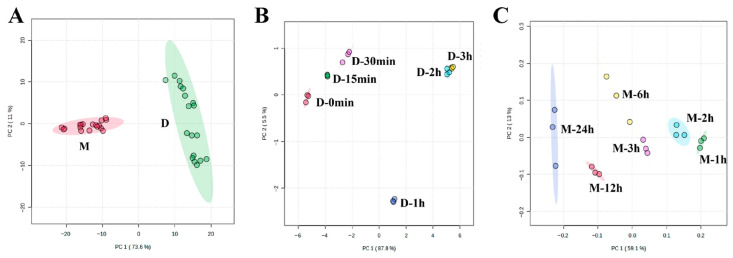
Score plot of PCA among the decocted (D) and macerated (M) samples (**A**). Score plot of PCA analysis among the decocted samples (**B**). Score plot of PCA analysis among the macerated samples (**C**).

PCA was employed to assess the global differences between decocted and macerated samples. The tight clustering of the QC samples in the PCA score plot (Figure S4) confirmed the high reproducibility and reliability of the dataset. As shown in Figure 2A, PC1 and PC2 explained 73.6% and 11.0% of the total variance, respectively. The clear separation of decocted and macerated groups along the PC1 axis indicates that this principal component primarily captures the compositional differences induced by the extraction method (heating or non-heating), which is the dominant source of variation. Subsequently, PCA was performed separately on the decocted (Figure 2B) and macerated sample sets (Figure 2C). In the decocted samples, PC1 and PC2 accounted for 87.6% and 5.4% of the total variance, respectively. The high variance captured by PC1 (87.6%) and the orderly shift in samples along the PC1 axis with increasing decoction time suggest that decoction duration is the primary factor driving chemical changes during this process, following a time-dependent trajectory. In the macerated samples, PC1 and PC2 accounted for 59.1% and 13.0%, respectively. Compared to the decocted samples (93.0%), the lower cumulative variance explained by PC1 and PC2 (72.1%) in the macerated samples suggests that the chemical changes during maceration are less strongly driven by processing time and more heterogeneous, likely due to the absence of heating that promotes uniform transformations. Nevertheless, the progressive shift in samples along the PC1 axis with increasing maceration time still indicates that extraction duration influences chemical composition even at room temperature. Quality control (QC) samples were tightly clustered in the PCA score plot (Figure S4), indicating good analytical stability and reproducibility of the UPLC-MS system.

To investigate the influence of processing time, one-way ANOVA with Benjamini–Hochberg false discovery rate (FDR) correction was performed on the macerated and decocted samples, respectively. The fold change (FC) was then calculated for each pair of groups. Differential metabolites were finally screened using the criteria of *p*(FDR) < 0.05 and FC > 2. In the decocted samples, 70 differential compounds were screened out, including 25 phenolic acids, 11 flavonoids, 15 iridoids, 5 amino acids, 3 nucleotides, 9 fatty acids, 1 organic acid and 1 other compound. Among them, 15 metabolites increased, 47 decreased, and 8 initially increased, then decreased. In the macerated samples, 35 differential compounds were found, comprising 5 phenolic acids, 8 flavonoids, 8 iridoids, 4 fatty acids, 1 amino acid, 6 nucleotides, 2 dipeptides and 1 organic acid. Of these, 11 metabolites increased, 14 decreased, and 10 exhibited an initial rise followed by a decline. Most differential metabolites were secondary metabolites, indicating that phenolic acids, flavonoids and iridoids were significantly influenced by different processing methods. A total of 22 selected differential metabolites and key compounds were quantitatively analyzed to provide more reliable evidence of their dynamic changes (Tables S5 and S6). For clarity, relative abundance was used to describe untargeted metabolomics results, whereas content was used only for the targeted quantitative analysis of representative compounds.

### 3.3. Metabolomic Difference During the Decoction Process

#### 3.3.1. Changes in Phenolic Acids

**Figure 3:**
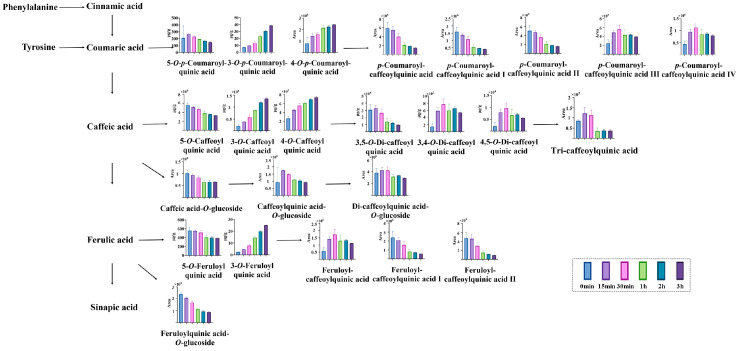
Distribution of phenolic acids in the decocted samples.

Phenolic acids, recognized as key quality markers for LJF in the Chinese pharmacopeia [35,36], are mainly derived from the shikimate pathway. In this pathway, tyrosine and phenylalanine are converted into coumaric acid and cinnamic acid, respectively. These intermediates subsequently undergo modifications such as esterification with quinic acid to form various substituted phenolic acid derivatives [37]. These compounds are widely associated with antioxidant capacity and contribute to the functional and sensory properties of LJF-derived beverages and food preparations [38]. With prolonged decoction time, phenolic acids showed significant changes (Figure 3). For mono-hydroxycinnamoyl quinic acids, the contents of 5-*O*-caffeoylquinic acid, 5-*O*-*p*-coumaroylquinic acid, and 5-*O*-feruloylquinic acid decreased over time. In contrast, the levels of 3-*O*-caffeoylquinic acid, 4-*O*-caffeoylquinic acid, 3-*O*-*p*-coumaroylquinic acid, 4-*O*-*p*-coumaroylquinic acid, and 3-*O*-feruloylquinic acid increased. It indicated that positional transformation might occur during decoction. Previous studies have reported that caffeoylquinic acid underwent acyl migration under thermal, alkaline, and acidic conditions [39,40]. The 5-hydroxycinnamoyl quinic acids tended to isomerize into their 3- and 4-substituted analogs. The trend observed in our study is consistent with these reports [41]. This observation may be related to thermally induced acyl migration, which could contribute to the interconversion of 5-*O*-substituted isomers into the more thermodynamically stable 3- and 4-*O*-substituted forms [42]. The progressive accumulation of 3-*O*- and 4-*O*-caffeoylquinic acids throughout decoction may suggest that thermal energy continuously drives this isomerization equilibrium toward the more stable isomers. These findings suggest that decoction exerted a pronounced influence on the accumulation of phenolic acids in LJF. Several representative phenolic acid derivatives with characteristic variation patterns were further quantified to illustrate their specific changes during decoction based on content levels.

Specifically, for caffeoylquinic acids, the content of 5-*O*-caffeoylquinic acid decreased from 55,754.68 μg/g at 0 min to 51,518.33, 46,650.83, 37,894.00, 35,628.47, and 32,783.05 μg/g at 15 min, 30 min, 1 h, 2 h, and 3 h, respectively. By contrast, the content of 3-*O*-caffeoylquinic acid increased from 2008.47 μg/g at 0 min to 3843.46, 5796.45, 8747.61, 11,902.05, and 13,576.29 μg/g over the same period, while that of 4-*O*-caffeoylquinic acid increased from 2611.33 μg/g to 4538.64, 5490.80, 6044.01, 6943.37, and 7329.63 μg/g, respectively. The content of 5-*O*-caffeoylquinic acid decreased to approximately 0.59-fold of its initial level, whereas 3-*O*- and 4-*O*-caffeoylquinic acids increased by approximately 6.76-fold and 2.81-fold, respectively, over the decoction period. For coumaroylquinic acids, the content of 5-*O*-*p*-coumaroylquinic acid began to decline after 15 min, decreasing from 265.01 μg/g at 15 min to 229.34, 193.85, 169.52, and 148.99 μg/g at 30 min, 1 h, 2 h, and 3 h, respectively. In comparison, the content of 3-*O*-*p*-coumaroylquinic acid increased from 7.30 μg/g at 0 min to 10.00, 13.29, 23.30, 30.76, and 38.80 μg/g at 15 min, 30 min, 1 h, 2 h, and 3 h, respectively, while the relative abundance of 4-*O*-*p*-coumaroylquinic acid increased continuously in the untargeted metabolomics analysis. The content of 5-*O-p*-coumaroylquinic acid decreased to approximately 0.56-fold of its level at 15 min, whereas 3-*O*-*p*-coumaroylquinic acid increased by approximately 5.32-fold compared to its initial level. Among feruloylquinic acids, the content of 5-*O*-feruloylquinic acid decreased from 560.34 μg/g at 0 min to 553.63, 515.27, 407.09, 402.36, and 388.95 μg/g at 15 min, 30 min, 1 h, 2 h, and 3 h, respectively, whereas the content of 3-*O*-feruloylquinic acid markedly increased from 2.48 μg/g at 0 min to 4.72, 7.86, 14.60, 19.98, and 25.10 μg/g at 15 min, 30 min, 1 h, 2 h, and 3 h, respectively. The content of 5-*O*-feruloylquinic acid showed a moderate decrease to approximately 0.69-fold of its initial level, whereas 3-*O*-feruloylquinic acid exhibited a marked increase of approximately 10.12-fold during decoction. Through the above results, 5-*O*-hydroxycinnamoyl quinic acids decreased to a relatively constant level after 30 min, whereas 3-*O*- and 4-*O*-hydroxycinnamoyl quinic acids showed a progressive increase over time. This variation pattern suggests that decoction may not only promote the extraction of chlorogenic acid analogs, but may also be accompanied by structural conversion among positional isomers. One possible explanation is that, under prolonged heating in an aqueous system, acyl migration may occur on the quinic acid scaffold, thereby contributing to the interconversion of 5-*O*-, 3-*O*-, and 4-*O*-hydroxycinnamoyl quinic acids.

For di-hydroxycinnamoyl quinic acids, the content of 3,5-*O*-di-caffeoylquinic acid showed a slight increase at 15 min, then decreased continuously, changing from 30,656.65 μg/g at 0 min to 32,467.48, 26,129.55, 13,945.35, 12,157.56, and 9535.57 μg/g at 15 min, 30 min, 1 h, 2 h, and 3 h, respectively. In contrast, the contents of 3,4-*O*-di-caffeoylquinic acid and 4,5-*O*-di-caffeoylquinic acid were markedly elevated during decoction compared with the initial time point. Specifically, the content of 3,4-*O*-di-caffeoylquinic acid increased from 1410.27 μg/g at 0 min to 5813.43, 7655.36, 5954.66, 6541.75, and 5368.61 μg/g at 15 min, 30 min, 1 h, 2 h, and 3 h, respectively, while that of 4,5-*O*-di-caffeoylquinic acid increased from 2043.42 μg/g to 7887.57, 9693.86, 6674.61, 7019.22, and 5528.85 μg/g over the same period. The content of 3,5-*O*-di-caffeoylquinic acid showed a continuous decline during decoction, decreasing to approximately 0.31-fold of its initial level at 3 h. By contrast, 3,4-*O*-di-caffeoylquinic acid and 4,5-*O*-di-caffeoylquinic acid increased markedly, reaching peak levels of approximately 5.43-fold and 4.74-fold of their initial contents at 30 min, respectively, and remained 3.81-fold and 2.71-fold higher than the initial levels at 3 h. However, both compounds showed a decline after 30 min, suggesting they were not stable during prolonged decoction. It has been reported that dicaffeoylquinic acids can degrade into caffeoylquinic acids, thereby promoting the accumulation of the latter [43]. Accordingly, the decline in di-caffeoylquinic acids observed after 30 min in the present study may be related to heat-induced degradation or conversion processes. In addition, the relative abundance of feruloyl-caffeoylquinic acid and *p*-coumaroyl caffeoylquinic acid isomers also exhibited distinct trends, which may be related to acyl migration during decoction. As for hydroxycinnamoyl quinic acid glucosides, the fold changes in caffeoylquinic acid-*O*-glucoside and di-caffeoylquinic acid-*O*-glucoside at 15 min were 1.91 and 1.12, respectively, relative to 0 min, but decreased after 15 min. The relative abundance of feruloylquinic acid-*O*-glucoside declined. The degradation of phenolic acid-*O*-glucoside may also contribute to the accumulation of hydroxycinnamoyl quinic acids [44]. The transient increase in caffeoylquinic acid-*O*-glucosides within the first 15 min, followed by a decrease, likely reflects a balance between extraction-driven release and heat-induced deglycosylation. Furthermore, the accumulation of 3-hydroxycinnamoyl quinic acids and 4-hydroxycinnamoyl quinic acids might arise from the degradation of di-caffeoylquinic acids and phenolic acid-*O*-glucosides. Di-caffeoylquinic acid has been reported to exhibit stronger uric acid-lowering activity and antioxidant capacity than mono-caffeoylquinic acid [45,46]. Considering the balance between constituent release and subsequent degradation or conversion, a decoction time of 15–30 min appears to be more suitable for preserving these characteristic phenolic constituents. From a functional perspective, these transformations may alter not only the overall phenolic profile but also the relative abundance of compounds with different antioxidant and bioactive properties, which is important for optimizing decoction conditions.

#### 3.3.2. Changes in Flavonoids

**Figure 4:**
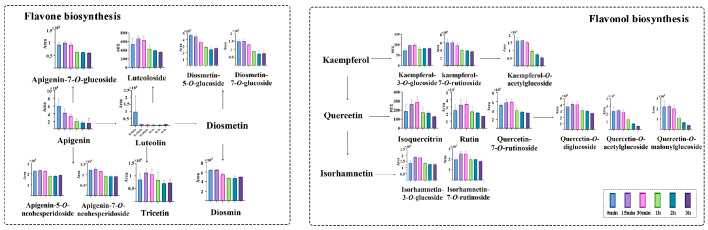
Distribution of flavonoids in the decocted samples.

There were 11 differential flavonoids identified, including apigenin, quercetin-*O*-malonylglucoside, quercetin-*O*-acetylglucoside, kaempferol-*O*-acetylglucoside, and diosmetin-7-*O*-glucoside. These flavonoids comprised both aglycones and their glycosidic derivatives. Apigenin was one of the predominant flavonoid aglycones in LJF, and its relative abundance decreased with prolonged decoction time. Specifically, the fold changes in apigenin at 15 min, 30 min, 1 h, 2 h, and 3 h were 0.70, 0.54, 0.34, 0.27, and 0.25, respectively, relative to the initial level (0 min). For the acylated flavonoid glucosides, the fold changes in kaempferol-*O*-acetylglucoside at the same time points were 1.01, 0.92, 0.59, 0.45, and 0.33, respectively, relative to 0 min. Similarly, the fold changes in quercetin-*O*-acetylglucoside were 1.04, 0.95, 0.55, 0.31, and 0.19, whereas those of quercetin-*O*-malonylglucoside were 1.02, 0.91, 0.51, 0.30, and 0.19, respectively. A marked reduction in the relative abundances of acylated flavonoid glucosides was observed after 30 min (Figure 4).

For non-acylated glycosidic forms of apigenin and luteolin, the *p*(FDR) values was below 0.05, but the FC was relatively modest (less than 2-fold). As illustrated in Figure 4, the flavone glycosides, including apigenin, luteolin, and diosmetin, were decreased after 30 min, with no significant changes observed from 1 to 3 h. Similarly, flavonol glycosides including quercetin, isorhamnetin, and kaempferol also declined after 30 min. Within the first 30 min, several non-acylated flavonoid glycosides showed slight increases. The contents of isoquercitrin and kaempferol-3-*O*-glucoside reached their highest levels at 30 min, increasing from 187.09 to 288.41 μg/g and from 71.87 to 98.29 μg/g, respectively, whereas luteoloside peaked at 15 min, increasing from 538.96 to 668.49 μg/g. Meanwhile, the relative abundances of isorhamnetin-3-*O*-glucoside, rutin and isorhamnetin-7-*O*-rutinoside also increased slightly within the first 30 min, showing fold changes of 1.31, 1.35 and 1.28, respectively, relative to their initial levels. The transient increase in certain flavonol glycosides within the first 30 min likely reflects a balance between extraction-driven release and concurrent thermal degradation. During the initial phase, the extraction rate exceeds the degradation rate, resulting in a net increase in detectable levels. However, as heating continues beyond 30 min, degradation becomes dominant, leading to a progressive decline. These findings collectively suggest that a 30 min decoction helps retain higher levels of flavonoids.

When comparing aglycones, non-acylated glycosides, and acylated glucosides, the glycosidic forms of apigenin and kaempferol exhibited greater heat resistance than their aglycone counterparts. The presence of glycosyl groups may increase resistance to thermal degradation, since cleavage of glycosidic bonds generally requires additional energy [47]. In contrast, acylated flavonoid glycosides appeared to be less stable than non-acylated glycosides during decoction, which may be related to the greater structural lability caused by acyl substitution under heating conditions. This aligns with previous reports indicating that kaempferol glycosides are significantly more stable than their acylated derivatives [48]. Overall, these results suggest that the thermal stability of flavonoids in LJF may be associated with their substitution patterns, and that a moderate decoction time may help retain glycosylated flavonoids while reducing the loss of structurally labile acylated derivatives. Such differences in thermal stability may further influence the retention of flavonoid-related bioactivities during decoction.

#### 3.3.3. Changes in Iridoids

**Figure 5:**
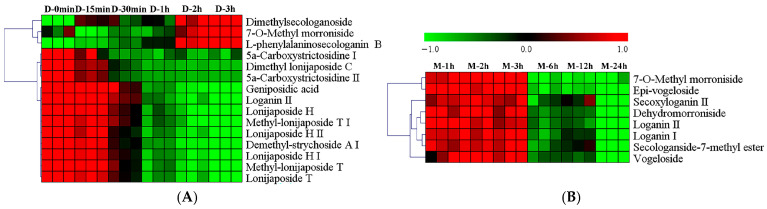
Heat map of differential iridoids in the decocted (**A**) and macerated samples (**B**).

Iridoids are important phytochemical constituents that have been associated with various health-related properties [49], including anti-inflammatory properties [50], and hypoglycemic activity [51]. Hierarchical clustering analysis was conducted on the 15 differential iridoids, which comprised 3 general iridoids, 9 nitrogen-containing seco-iridoids and 3 seco-iridoids, accounting for 13.04%, 56.25% and 11.11% of their respective subclasses. This distribution suggests that nitrogen-containing seco-iridoids may be more sensitive to thermal treatment [52]. Since iridoids have been reported to be prone to hydrolysis, degradation, and structural transformation during thermal processing [53], the marked decrease observed in the present study may indicate the relatively limited thermal stability of nitrogen-containing seco-iridoids. As shown in Figure 5A, three iridoids showed an upward trend in content, including one general iridoid (7-*O*-methyl morroniside) and two seco-iridoids (dimethylsecologanoside, L-phenylalaninosecologanin B). The relative abundance of L-phenylalaninosecologanin B increased markedly over time, with fold changes of 2.11, 3.23, 4.61, 8.32, and 11.17 at 15 min, 30 min, 1 h, 2 h, and 3 h, respectively, relative to 0 min. In contrast, 12 iridoids showed decreased relative abundance, including two general iridoids, one seco-iridoid and nine nitrogen-containing seco-iridoids. Among the general iridoids, the relative abundance of loganin II declined with fold changes of 0.89, 0.80, 0.62, 0.55, and 0.46 at the corresponding time points, while geniposidic acid showed fold changes of 0.99, 0.85, 0.59, 0.52, and 0.46. Among the nitrogen-containing seco-iridoids, the fold changes in lonijaposide T over the same intervals were 0.70, 0.45, 0.21, 0.13, and 0.05, respectively, while those of lonijaposide H were 0.84, 0.61, 0.39, 0.26, and 0.19, respectively. Nitrogen-containing seco-iridoids exhibited the most pronounced degradation. Therefore, it is speculated that the presence of a nitrogen-containing moiety may increase chemical reactivity and reduce thermal stability, thereby making these compounds more susceptible to degradation or structural transformation under prolonged heating. These results indicate that most iridoids decreased rapidly after 30 min of decoction, while a few increased substantially. Together, they show that decoction time plays an important role in shaping the iridoid composition of LJF. In particular, the marked decrease in several heat-sensitive iridoids, especially nitrogen-containing seco-iridoids, indicates that prolonged decoction reduces the levels of these characteristic constituents. Therefore, careful control of decoction duration may be important for better preserving these characteristic compounds. To preserve higher levels of most iridoids, a moderate decoction time of 15–30 min appears appropriate.

### 3.4. Metabolomic Difference During Maceration Process

#### 3.4.1. Changes in Phenolic Acids

**Figure 6:**
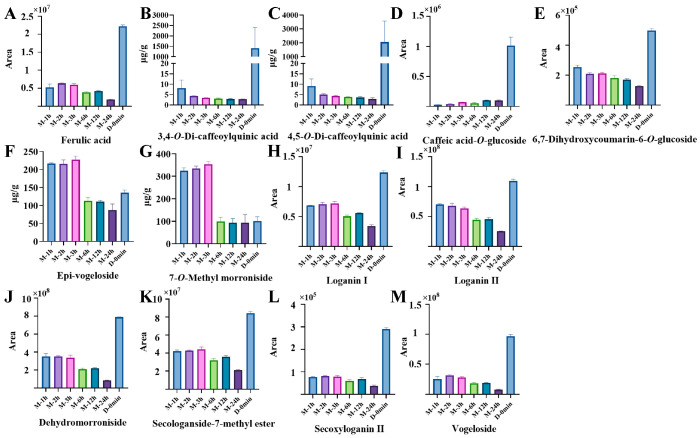
The content of differential phenolic acids and iridoids during the maceration process (1 h, 2 h, 3 h, 6 h, 12 h, and 24 h) and the decoction process (0 min): (**A**) Ferulic acid; (**B**) 3,4-*O*-di-caffeoylquinic acid; (**C**) 4,5-*O*-di-caffeoylquinic acid; (**D**) Caffeic acid-*O*-glucoside; (**E**) 6,7-Dihydroxycoumarin-6-*O*-glucoside; (**F**) Epi-vogeloside; (**G**) 7-*O*-Methyl morroniside; (**H**) Loganin I; (**I**) Loganin II; (**J**) Dehydromorroniside; (**K**) Secologanoside-7-methyl ester; (**L**) Secoxyloganin II; (**M**) Vogeloside.

During the maceration process, five phenolic acids were identified as differential compounds, including ferulic acid, 3,4-*O*-di-caffeoylquinic acid, 4,5-*O*-di-caffeoylquinic acid, caffeic acid-*O*-glucoside and 6,7-dihydroxycoumarin-6-*O*-glucoside. The relative abundance of ferulic acid increased from 1 h to 2 h, and then decreased. However, its content during maceration remained substantially lower than during decoction. Specifically, the fold change in ferulic acid in the decocted sample at 0 min was 3.50 relative to that in the macerated sample at 2 h (Figure 6A). This trend likely reflects a balance between extraction-driven release and slow degradation under cold-maceration conditions, consistent with previous reports that ferulic acid degradation is temperature-dependent [53] and that cold-soak conditions alter phenolic extraction kinetics [54]. Mono-caffeoylquinic acids, feruloylquinic acids and coumaroylquinic acids exhibited no significant changes during the maceration process. Moreover, the levels of mono-caffeoylquinic acids and mono-feruloylquinic acids in the macerated samples were considerably lower than those in the decocted samples. From 1 h to 24 h of maceration, the content of 5-*O*-caffeoylquinic acid increased slightly from 91.68 to 106.41 μg/g at 1–3 h, and then decreased to 65.67 μg/g at 24 h. Meanwhile, the content of 3-*O*-caffeoylquinic acid changed from 4.96 to 5.34 μg/g, and that of 4-*O*-caffeoylquinic acid changed from 4.48 to 3.59 μg/g, respectively. In comparison, the contents of 3-*O*-, 4-*O*-, and 5-*O*-caffeoylquinic acids at 0 min in the decocted samples were 2008.47, 2611.33, and 55,754.68 μg/g, respectively, indicating that their levels in macerated samples remained markedly lower than those in decocted samples (Figure S5A–C). Similarly, from 1 h to 24 h of maceration, the contents of 3-*O*-feruloylquinic acid and 5-*O*-feruloylquinic acid increased from 0.34 to 0.64 μg/g and from 48.04 to 59.81 μg/g, respectively. By comparison, their contents at 0 min in the decocted samples were 2.48 and 560.34 μg/g, respectively, again showing lower overall levels in the macerated samples. In contrast, mono-coumaroylquinic acids showed only minor differences between decoction and maceration (Figure S5D,E). In contrast, mono-coumaroylquinic acids showed no notable differences between maceration processes.

Regarding di-hydroxycinnamoyl quinic acids, the contents of 3,4-*O*-di-caffeoylquinic acid, 3,5-*O*-di-caffeoylquinic acid and 4,5-*O*-di-caffeoylquinic acid gradually decreased with prolonged maceration time. Specifically, the content of 3,4-*O*-di-caffeoylquinic acid decreased from 8.08 μg/g at 1 h to 4.31, 3.44, 3.09, 2.86 and 2.75 μg/g at 2 h, 3 h, 6 h, 12 h and 24 h, respectively. The content of 4,5-*O*-di-caffeoylquinic acid decreased from 9.05 μg/g to 4.98, 4.30, 3.96, 3.67 and 2.96 μg/g over the same intervals. The contents of 3,4-*O*-di-caffeoylquinic acid and 4,5-*O*-di-caffeoylquinic acid at 0 min in the decocted samples remained substantially higher than those observed throughout the maceration process; specifically, their contents reached 1410.27 and 2043.42 μg/g, respectively, indicating that these compounds were maintained at markedly lower levels in the macerated samples. (Figure 6B,C). As for caffeic acid-*O*-glucoside, its relative abundance showed an upward trend, with fold changes of 1.45, 2.71, 2.05, 3.89, and 3.62 at 2 h, 3 h, 6 h, 12 h, and 24 h, respectively, relative to the 1 h initial value. Conversely, the relative abundance of 6,7-dihydroxycoumarin-6-*O*-glucoside decreased over time, with fold changes of 0.83, 0.84, 0.72, 0.67, and 0.50, respectively, relative to the level at 1 h. The levels of these phenolic acid glucosides were also lower than those observed in the decoction process (Figure 6D,E). Overall, the substantially lower levels of phenolic acids in soaked samples compared to decocted samples reflect the critical role of thermal energy in accelerating molecular diffusion and promoting the release of bound phenolic acids, highlighting the superior extraction efficiency of decoction for phenolic compounds.

#### 3.4.2. Changes in Flavonoids

A total of 8 differential flavonoids were identified, including apigenin-7-*O*-glucoside, luteoloside, quercetin-*O*-diglucoside, diosmetin-5-*O*-glucoside, diosmetin-7-*O*-glucoside, etc.

Among flavone glycosides, the content of luteoloside gradually decreased with the maceration time (Figure S6). Specifically, its content decreased from 5.4 μg/g at 1 h to 2.5, 1.64, 1.28, 1.43, and 0.85 μg/g at 2 h, 3 h, 6 h, 12 h, and 24 h, respectively. In comparison, the level of luteoloside in the 0 min decocted sample was markedly higher than that observed in the macerated samples. Similarly, the relative abundances of apigenin-7-*O*-glucoside, diosmetin-5-*O*-glucoside and diosmetin-7-*O*-glucoside decreased with the maceration time. Their relative abundances in the decocted sample at 0 min showed fold changes of 4.24, 4.99, and 3.13, respectively, compared with those in the macerated sample at 1 h. The continuous decline of these flavone glycosides from the earliest time points suggests they are initially extractable at room temperature but undergo slow degradation or conversion during prolonged maceration.

Both acylated and non-acylated flavonol glycosides showed an initial increase followed by a decrease during maceration, with peak relative abundance occurring at 6 h or 12 h. For instance, kaempferol-7-*O*-rutinoside and quercetin-*O*-diglucoside reached their maximum levels at 6 h, showing fold changes of 2.06 and 2.70, respectively, relative to 1 h. This transient increase reflects a balance between extraction-driven release and concurrent degradation, with extraction outpacing degradation in the early phase, followed by a net decline as degradation becomes dominant. Nevertheless, their relative abundances were still substantially lower than those in the decocted samples. Their relative abundances in the decocted samples at 0 min showed fold changes of 16.86 and 78.57, respectively, relative to those in the macerated samples at 6 h. The relative abundance of kaempferol-*O*-acetylglucoside reached its maximum at 12 h, with a fold change of 2.03 relative to that at 1 h. This later peak may reflect slower extraction kinetics due to its acylated structure. Compared with the macerated sample at 12 h, the fold change in kaempferol-*O*-acetylglucoside in the decocted sample at 0 min was 8.22. Overall, the levels of flavonoids in the decoction process were significantly higher than in the maceration process, indicating that heating also facilitates flavonoid release [18]. However, compared with phenolic acids, the promoting effect of decoction was less pronounced for most flavonoids. This suggests that a larger proportion of flavonoids may exist in free or weakly bound forms that are extractable without heating, whereas phenolic acids are predominantly in bound forms requiring thermal energy for release [55].

#### 3.4.3. Changes in Iridoids

Hierarchical cluster analysis was performed on the eight differential iridoids, which consisted of four general iridoids and four seco-iridoids. As shown in Figure 5B, the levels of these iridoids decreased significantly after 3 h of maceration, and most accumulated gently from 1 h to 3 h. The general iridoids included two loganin isomers, 7-*O*-methyl morroniside and dehydromorroniside. Specifically, the content of 7-*O*-methyl morroniside increased from 324.56 μg/g at 1 h to 354.59 μg/g at 3 h, and then decreased to 99.65, 92.97 and 92.91 μg/g at 6 h, 12 h and 24 h, respectively. The relative abundances of loganin I, loganin II, and dehydromorroniside decreased over time, showing fold changes of 0.47, 0.40, and 0.25, respectively, at 24 h relative to 3 h. The seco-iridoids, including secoxyloganin II, epi-vogeloside, secologanside-7-methyl ester and vogeloside, also exhibited reductions, showing fold changes ranging from 0.27 to 0.48 at 24 h compared with 3 h. The content of epi-vogeloside was 216.81, 216.04, and 227.87 μg/g at 1 h, 2 h, and 3 h, respectively, but decreased to 112.54, 110.71, and 87.36 μg/g at 6 h, 12 h, and 24 h. Compared with decocted samples, the contents of epi-vogeloside and 7-*O*-methyl morroniside in the first 3 h of macerated samples were higher, reaching 1.68- and 3.51-fold of the values at 0 min in the decocted samples, respectively (Figure 6F,G). This observation indicates that these two compounds are thermally labile; during decoction, they undergo rapid degradation, whereas in cold maceration they are preserved. In contrast, the other six differential iridoids were present at lower levels in soaked samples (Figure 6H–M). Specifically, compared with the macerated samples at 3 h, the relative abundances of loganin I, loganin II, dehydromorroniside, secologanside-7-methyl ester, secoxyloganin II and vogeloside at showed fold changes of 1.72, 1.72, 2.35, 1.90, 3.67, and 3.44, respectively. Notably, these ratios were considerably lower than those observed for phenolic acids and flavonoids. Furthermore, no nitrogen-containing seco-iridoids were identified as differential compounds during maceration, indicating that this class of compounds is particularly sensitive to thermal treatment. This suggests that iridoids are more readily extractable under maceration conditions compared to phenolic acids and flavonoids, possibly because a larger proportion of iridoids exists in free or weakly bound forms. This interpretation is supported by the observation that no nitrogen-containing seco-iridoids were identified as differential compounds during maceration, indicating that this class remains stable under room temperature conditions but is highly sensitive to thermal degradation.

The dynamic chemical changes observed during decoction and maceration have direct implications for the application of LJF. Compared with decoction, maceration yielded substantially lower extraction yields for most bioactive compounds, particularly phenolic acids and flavonoids. A notable observation was that certain iridoids exhibited higher levels in macerated samples during the first 3 h than in decocted samples. This suggests that these thermally labile compounds are partially degraded during decoction, whereas maceration preserves them, highlighting maceration’s potential to retain heat-sensitive bioactive components. From a functional application perspective, the choice between methods should be guided by the intended application. Decoction achieves substantially higher yields of phenolic acids and flavonoids, making it suitable for products with high biological activity [56]. However, the degradation of di-caffeoylquinic acids and nitrogen-containing seco-iridoids after 30 min supports a decoction window of 15–30 min to preserve these bioactive components. In contrast, maceration favors the retention of heat-sensitive iridoids while reducing the extraction of phenolic acids potentially associated with bitterness [57]. This maceration-based preparation may be more suitable for applications requiring milder extraction conditions or lower levels of bioactive components, such as cold-brew teas and functional beverages. These findings provide a scientific basis for optimizing LJF preparation according to the desired chemical profile and application.

## 4. Conclusions

A comprehensive investigation of LJF was conducted to examine dynamic changes in its chemical composition during maceration and decoction. A total of 70 and 35 differential compounds were identified in the decocted and macerated samples, respectively. Phenolic acids, flavonoids, and iridoids were found to be markedly influenced by the preparation method. Both the content and variety of phenolic acids and flavonoids differed significantly between the macerated and decocted samples, with considerably lower levels observed in the macerated samples. Quantitative analysis indicated that representative phenolic acids remained at much lower levels during maceration; for example, 5-*O*-caffeoylquinic acid was only 65.67 μg/g at 24 h of maceration, whereas it remained 32,783.05 μg/g after 3 h of decoction. In a similar pattern, luteoloside showed a much higher level under decoction, reaching 357.81 μg/g after 3 h, whereas only 0.85 μg/g was detected after 24 h of maceration. In contrast, the decoction process significantly enhanced the release of these compounds. Regarding phenolic acids, the concentrations of 5-hydroxycinnamoyl quinic acids, di-hydroxycinnamoyl quinic acids, and their glucosides decreased over time, whereas those of 3- and 4-hydroxycinnamoyl quinic acids increased. During decoction, 5-hydroxycinnamoyl quinic acids, di-hydroxycinnamoyl quinic acids, and their glucosides generally decreased over time, whereas 3- and 4-hydroxycinnamoyl quinic acids increased, suggesting that isomeric transformation of hydroxycinnamoyl quinic acids may occur under thermal conditions. As for flavonoids, their overall content declined, with non-acylated flavonoid glycosides exhibiting greater stability than their acylated counterparts and aglycones. Among the iridoids, nitrogen-containing seco-iridoids underwent more extensive degradation compared to other iridoids. The majority of differential secondary metabolites decreased after 15–30 min of decoction, while a small fraction increased with prolonged heating. By comparing the magnitude of variations, mono-caffeoylquinic acids, di-caffeoylquinic acids, apigenin and acylated flavonoid glucosides, together with nitrogen-containing seco-iridoids, were susceptible to decoction time. These dynamic variations in the metabolite profile throughout the preparation process underlie the differences in biological activities.

Overall, these findings indicate that the chemical transformations occurring during decoction may have important implications for the bioactivity, functional food utilization, and traditional preparation of LJF. Appropriate control of decoction conditions may help improve the retention of characteristic phytochemicals, thereby supporting the more rational and standardized use of LJF in both medicinal and edible applications. Therefore, precise monitoring and control of chemical profiles provide a critical chemical rationale for ensuring more consistent efficacy and more reliable utilization of this medicinal and edible material. However, some limitations should also be acknowledged. In the present study, absolute quantification was performed only for selected representative compounds, and a broader quantitative evaluation of additional constituents remains to be performed. Moreover, the proposed mechanisms (e.g., acyl migration, hydrolysis) are inferred from indirect evidence and literature. These pathways should be further validated via targeted degradation experiments.

## Data Availability

The original contributions presented in the study are included in the article/Supplementary Materials. Further inquiries can be directed to the corresponding author.

## Supplementary Materials

The following supporting information can be downloaded at: https://www.mdpi.com/article/10.3390/foods15081421/s1, Table S1: Information on reference standards for quantitative analysis; Table S2: Calibration curve parameters of the analyzed compounds; Table S3: Secondary metabolites identified in the processed LJF samples; Table S4: Primary metabolites identified in the processed LJF samples; Table S5: Contents of 22 compounds in decocted samples (mean ± SD, *n* = 3); Table S6: Contents of 22 compounds in macerated samples (mean ± SD, *n* = 3); Figure S1: The peak number and accumulate peak area of repeatability (A), precision (B,C), stability (D) in different RSD ranges; Figure S2: The molecular networking for LJF; Figure S3: Proposed fragmentation pathways of representative iridoids (A), phenolic acids (B) and flavonoids (C) based on MS/MS analysis; Figure S4: Score plot of PCA among the QC samples, different decoction times (D) and different maceration times (M) samples; Figure S5: The content of phenolic acids during maceration process (1 h, 2 h, 3 h, 6 h, 12 h and 24 h) and decoction process (0 min); Figure S6: Distribution of flavonoids in the macerated samples.
