# Supplementary material for: Dynamic Chemical Profiling of Lonicera japonica Flos During the Maceration and Decoction Processes Integrating UPLC-MS and Molecular Networking
Source: Foods. 2026 Apr 19;15(8):1421. doi: 10.3390/foods15081421 (PMC13114655; doi:10.3390/foods15081421)
Supplement: Supplementary file 1 [file foods-15-01421-s001.zip › foods-4205133-supplementary.pdf]

Dynamic chemical profiling of *Lonicera japonica* Flos during the maceration and decoction processes integrating UPLC-MS and molecular networking

Hui Ding[1], Chenglong Sun[1], Chuanzhi Kang[2], Yuemeng Liu[1], Xiao Wang[1], Lili Li[1]*

1 Shandong Engineering Research Center for Innovation and Application of General Technology for Separation of Natural Products, Shandong Analysis and Test Center, Qilu University of Technology (Shandong Academy of Sciences), Jinan, 250014, China

2 State Key Laboratory for Quality Ensurance, and Sustainable Use of Dao-di Herbs, National Resource Center for Chinese Materia Medica, China Academy of Chinese Medical Sciences, Beijing, 100700, China

Method validation

Repeatability was assessed using six replicate QC samples. The relative standard deviation (RSD) was calculated for each peak in six QC samples. Results indicated that 98.60 % of the peaks had an RSD below 20%, accounting for 99.24% of the total peak area (Figure S1A), indicating the good repeatability of the method.

Precision was evaluated both within a single day and across multiple days. Intra-day precision was evaluated by analyzing QC samples six times within a single day, with 97.63% of peaks showing an RSD < 20%, representing 99.26% of the total peak area (Figure S1B). For inter-day precision, 18 QC samples were analyzed over three consecutive days, and 97.39% of the peaks exhibited an RSD < 20%, accounting for 96.01% of the total peak area (Figure S1C). These results confirm the high precision of the method.

Stability was examined over 48 hours by analyzing QC samples at 6-hour intervals. Here, 93.49% of peaks showed an RSD below 20%, constituting 97.11% of the total peak area (Figure S1D), indicating good sample stability within this period. These results indicated that the LC-MS method had satisfactory repeatability, precision, and stability for metabolomics analysis.

For the subsequent quantitative analysis of selected compounds, linearity was further evaluated using authentic reference standards. Calibration curves were constructed by plotting peak area versus concentration. For analytes with similar concentration ranges in both soaked and decocted samples, a single calibration curve was applied. For analytes showing marked concentration differences between the two extraction methods, separate low- and high-concentration calibration curves were established using the same reference standards. The low-range curves were used for the soaked samples, whereas the high-range curves were used for the decocted samples. All target analytes showed good linear relationships within the tested concentration ranges, and the measured concentrations of all analytes fell within the corresponding validated linear ranges (Table S2), supporting the reliability of the quantitative results.

Table S1 Information on reference standards for quantitative analysis.

NO.	Compound	Purity	Supplier
1	3-*O*-Caffeoylquinic acid	purity ≥ 98%	Chengdu Desite Biotechnology Co., Ltd.
2	4-*O*-Caffeoylquinic acid	purity ≥ 98%	Chengdu Desite Biotechnology Co., Ltd.
3	5-*O*-Caffeoylquinic acid	purity ≥ 98%	Chengdu Desite Biotechnology Co., Ltd.
4	3-*O*-Feruloylquinic acid	purity ≥ 98%	Chengdu Desite Biotechnology Co., Ltd.
5	5-*O*-Feruloylquinic acid	purity ≥ 98%	Chengdu Desite Biotechnology Co., Ltd.
6	3-*O*-*p*-Coumaroylquinic acid	purity ≥ 98%	Jiangsu Yongjian Biotechnology Co., Ltd.
7	5-*O*-*p*-Coumaroylquinic acid	purity ≥ 98%	Jiangsu Yongjian Biotechnology Co., Ltd.
8	3,4-*O*-Dicaffeoylquinic acid	purity ≥ 98%	Shanghai Yuanye Bio-Technology Co., Ltd.
9	3,5-*O*-Dicaffeoylquinic acid	purity ≥ 98%	Shanghai Yuanye Bio-Technology Co., Ltd.
10	4,5-*O*-Dicaffeoylquinic acid	purity ≥ 98%	Shanghai Yuanye Bio-Technology Co., Ltd.
11	Luteoloside	purity ≥ 98%	Shanghai Yuanye Bio-Technology Co., Ltd.
12	Kaempferol-3-*O*-glucoside	purity ≥ 98%	Shanghai Yuanye Bio-Technology Co., Ltd.
13	Isoquercitrin	purity ≥ 98%	Shanghai Yuanye Bio-Technology Co., Ltd.
14	Loganic acid	purity ≥ 98%	Chengdu Desite Biotechnology Co., Ltd.
15	Secologanoside	purity ≥ 98%	Sichuan Weikeqi Biological Technology Co., Ltd.
16	Morroniside	purity ≥ 98%	Sichuan Weikeqi Biological Technology Co., Ltd.
17	Epi-vogeloside	purity ≥ 98%	Chengdu Desite Biotechnology Co., Ltd.
18	Loganin	purity ≥ 98%	Chengdu Desite Biotechnology Co., Ltd.
19	(E)-Aldosecologanin	purity ≥ 98%	Sichuan Weikeqi Biological Technology Co., Ltd.
20	(Z)-Aldosecologanin	purity ≥ 98%	Sichuan Weikeqi Biological Technology Co., Ltd.
21	Sweroside	purity ≥ 98%	Chengdu Desite Biotechnology Co., Ltd.
22	7-*O*-Methyl morroniside	purity ≥ 98%	Chengdu Desite Biotechnology Co., Ltd.

Table S2. Calibration curve parameters of the analyzed compounds.

NO.	Name	Calibration curve	R^2	Linear range (ng/mL)	Applied samples
1	3-*O*-Caffeoylquinic acid	y = 0.0004x - 8.1785	0.9997	25-1000	Macerated
		y = 0.001x - 19027	0.9928	5000-200000	Decocted
2	4-*O*-Caffeoylquinic acid	y = 0.0003x - 61.651	0.9963	25-2500	Macerated
		y = 0.0005x - 2309.6	0.9996	5000-100000	Decocted
3	5-*O*-Caffeoylquinic acid	y = 0.0006x - 315.07	0.9944	100-10000	Macerated
		y = 0.0024x - 128995	0.9946	50000-600000	Decocted
4	3-*O*-*p*-Coumaroylquinic acid	y = 0.0003x - 11.092	0.9994	25-1000	Both
5	5-*O*-*p*-Coumaroylquinic acid	y = 0.0004x - 245.76	0.9963	50-10000	Both

6	3-*O*-Feruloylquinic acid	y = 0.0002x + 0.1358	0.9995	2.5-500	Both
7	5-*O*-Feruloylquinic acid	y = 0.0004x - 245.92	0.9948	25-10000	Both
8	3,4-*O*-Di-caffeoylquinic acid	y = 0.0004x - 20.676	0.9965	25-500	Macerated
		y = 0.0009x - 7551.5	0.9921	2500-100000	Decocted
9	3,5-*O*-Di-caffeoylquinic acid	y = 0.0018x - 74.217	0.9973	25-2500	Macerated
		y = 0.0024x + 686.08	0.9999	20000-400000	Decocted
10	4,5-*O*-Di-caffeoylquinic acid	y= 0.0002x - 7.9718	0.9994	25-500	Macerated
		y = 0.0008x - 12846	0.9915	2500-200000	Decocted
11	Luteoloside	y = 0.0002x - 2.5337	0.9995	5-100	Macerated
		y = 0.0006x - 854.89	0.9934	250-10000	Decocted
12	Isoquercitrin	y = 0.0001 x - 0.3428	0.9973	2.5-100	Macerated
		y = 0.0002x - 347.93	0.9924	100-5000	Decocted
13	Kaempferol-3-*O*-glucoside	y = 0.0004x - 28.378	0.9969	25-1000	Both
14	Loganic acid	y = 0.0007x - 2368.3	0.9963	1000-50000	Both
15	Secologanoside	y = 0.0005x - 1845.4	0.9992	2500-50000	Both
16	Morroniside	y = 0.003x - 170.32	0.9954	5000-100000	Both
17	Epi-vogeloside	y = 0.0003x - 151.64	0.9985	100-5000	Both
18	Loganin	y = 0.0005x - 2343.6	0.9956	2500-50000	Both
19	(E)-Aldosecologanin	y = 0.0048x - 24655	0.9949	10000-200000	Both
20	(Z)-Aldosecologanin	y = 0.0044x - 3480.3	0.998	1000-100000	Both
21	Sweroside	y = 0.0003x - 543.78	0.9934	250-20000	Both
22	7-*O*-Methyl morroniside	y = 0.0488x - 173.23	0.9931	500-10000	Both

Table S3 Secondary metabolites identified in the processed LJF samples.

NO.	Name	t_R/min	m/z	Formula	Classification	Mode	MS Fragments	Identification
1	3-*O*-Caffeoylquinic acid	4.2	353.0867	$C_{16}H_{18}O_9$	Phenolic acids	$[M-H]^-$	191, 179, 135	Level 1
2	5-*O*-Caffeoylquinic acid	7.3	353.0867	$C_{16}H_{18}O_9$	Phenolic acids	$[M-H]^-$	191, 179, 163, 135	Level 1
3	4-*O*-Caffeoylquinic acid	8.0	353.0867	$C_{16}H_{18}O_9$	Phenolic acids	$[M-H]^-$	191, 179, 135	Level 1
4	3-*O*-*p*-Coumaroylquinic acid	6.2	337.0925	$C_{16}H_{18}O_8$	Phenolic acids	$[M-H]^-$	191, 163	Level 1
5	5-*O*-*p*-Coumaroylquinic acid	10.2	337.0925	$C_{16}H_{18}O_8$	Phenolic acids	$[M-H]^-$	191, 173, 163	Level 1
6	4-*O*-*p*-Coumaroylquinic acid	10.5	337.0925	$C_{16}H_{18}O_8$	Phenolic acids	$[M-H]^-$	191, 173, 163	Level 1
7	3-*O*-Feruloylquinic acid	7.7	367.1039	$C_{17}H_{20}O_9$	Phenolic acids	$[M-H]^-$	193, 173	Level 1
8	5-*O*-Feruloylquinic acid	12.0	367.1039	$C_{17}H_{20}O_9$	Phenolic acids	$[M-H]^-$	193, 191, 173	Level 1
9	4-*O*-Feruloylquinic acid	13.4	367.1039	$C_{17}H_{20}O_9$	Phenolic acids	$[M-H]^-$	193, 191, 173, 93	Level 1
10	3,4-*O*-Di-caffeoylquinic acid	16.3	515.1196	$C_{25}H_{24}O_{12}$	Phenolic acids	$[M-H]^-$	353, 191, 179, 173	Level 1
11	3,5-*O*-Di-caffeoylquinic acid	16.8	515.1196	$C_{25}H_{24}O_{12}$	Phenolic acids	$[M-H]^-$	353, 191, 179, 173	Level 1
12	4,5-*O*-Di-caffeoylquinic acid	17.7	515.1196	$C_{25}H_{24}O_{12}$	Phenolic acids	$[M-H]^-$	353, 191, 179, 173	Level 1
13	Hydroxybenzoic-*O*-glucoside	4.2	299.0772	$C_{13}H_{16}O_8$	Phenolic acids	$[M-H]^-$	137, 93	Level 2[a]
14	Hydroxybenzoic-*O*-glucoside I	5.6	299.0772	$C_{13}H_{16}O_8$	Phenolic acids	$[M-H]^-$	137, 94, 93	Level 2[a]
15	Hydroxybenzoic acid	15.0	137.0235	$C_7H_6O_3$	Phenolic acids	$[M-H]^-$	94, 93	Level 2[a]
16	Hydroxybenzoic acid I	5.0	137.0235	$C_7H_6O_3$	Phenolic acids	$[M-H]^-$	93	Level 2[a]
17	Protocatechuic acid	3.2	153.0183	$C_7H_6O_4$	Phenolic acids	$[M-H]^-$	109, 108	Level 2[a]
18	Trans-4-Coumaric acid	11.5	163.0393	$C_9H_8O_3$	Phenolic acids	$[M-H]^-$	119	Level 2[a]
19	Isoeugenol	20.2	163.0755	$C_{10}H_{12}O_2$	Phenolic acids	$[M-H]^-$	148	Level 2[a]
20	Vanillic acid	3.0	167.0340	$C_8H_8O_4$	Phenolic acids	$[M-H]^-$	152, 123, 108	Level 2[a]
21	Quinic acid	0.8	191.0558	$C_7H_{12}O_6$	Phenolic acids	$[M-H]^-$	137	Level 1
22	Syringic acid	4.2	197.0449	$C_9H_{10}O_5$	Phenolic acids	$[M-H]^-$	123	Level 2[a]
23	Protocatechuic acid-4-*O*-glucoside	2.5	315.0717	$C_{13}H_{16}O_9$	Phenolic acids	$[M-H]^-$	153	Level 1
24	*p*-Coumaroyl-glucoside	7.3	325.0921	$C_{15}H_{18}O_8$	Phenolic acids	$[M-H]^-$	163	Level 2[a]
25	*p*-Coumaroyl-glucoside I	14.6	325.0929	$C_{15}H_{18}O_8$	Phenolic acids	$[M-H]^-$	163	Level 2[a]
26	Caffeic acid-*O*-glucoside	6.5	341.0879	$C_{15}H_{18}O_9$	Phenolic acids	$[M-H]^-$	179, 135	Level 2[a]
27	Demethyl-vanillic acid	19.7	419.0982	$C_{20}H_{20}O_{10}$	Phenolic acids	$[M-H]^-$	152, 108	Level 2[a]
28	*p*-Coumaroyl caffeoylquinic acid	18.4	499.1249	$C_{25}H_{24}O_{11}$	Phenolic acids	$[M-H]^-$	353, 337, 191, 179	Level 2[b]
29	*p*-Coumaroyl caffeoylquinic acid I	18.6	499.1249	$C_{25}H_{24}O_{11}$	Phenolic acids	$[M-H]^-$	353, 337, 191, 179	Level 2[b]
30	*p*-Coumaroyl caffeoylquinic acid II	18.9	499.1249	$C_{25}H_{24}O_{11}$	Phenolic acids	$[M-H]^-$	353, 337, 191, 179	Level 2[c]
31	*p*-Coumaroyl caffeoylquinic acid III	19.4	499.1249	$C_{25}H_{24}O_{11}$	Phenolic acids	$[M-H]^-$	353, 337, 191,179	Level 2[b]
32	*p*-Coumaroyl caffeoylquinic acid IV	19.6	499.1249	$C_{25}H_{24}O_{11}$	Phenolic acids	$[M-H]^-$	353, 337, 191, 179	Level 2[c]
33	Caffeoylquinic acid-*O*-glucoside	4.0	515.1404	$C_{22}H_{28}O_{14}$	Phenolic acids	$[M-H]^-$	353, 191, 179, 173	Level 2[b]
34	Feruloylcaffeoylquinic acid	19.2	529.1354	$C_{26}H_{26}O_{12}$	Phenolic acids	$[M-H]^-$	367, 353, 191, 173	Level 2[b]
35	Feruloylcaffeoylquinic acid I	19.6	529.1354	$C_{26}H_{26}O_{12}$	Phenolic acids	$[M-H]^-$	367, 353, 191, 179, 173	Level 2[b]

36	Feruloylcaffeoylquinic acid II	20.0	529.1354	$C_{26}H_{26}O_{12}$	Phenolic acids	[M-H]$^-$	367, 353, 191, 179	Level 2[c]
37	Feruloylquinic acid-*O*-glucoside	5.8	529.1567	$C_{23}H_{30}O_{14}$	Phenolic acids	[M-H]$^-$	367, 193	Level 2[b]
38	Di-caffeoylquinic acid-*O*-glucoside	13.7	677.1712	$C_{31}H_{34}O_{17}$	Phenolic acids	[M-H]$^-$	515, 353, 191, 179	Level 2[c]
39	Di-caffeoylquinic acid-*O*-glucoside I	14.4	677.1712	$C_{31}H_{34}O_{17}$	Phenolic acids	[M-H]$^-$	515, 353, 191, 179, 173	Level 2[c]
40	Tri-caffeoylquinic acid	20.6	677.1504	$C_{34}H_{30}O_{15}$	Phenolic acids	[M-H]$^-$	515, 353, 191, 179, 173	Level 2[b]
41	Cinnamic acid	24.0	149.0597	$C_9H_8O_2$	Phenolic acids	[M+H]$^+$	131, 103	Level 1
42	Methyl trans-cinnamic acid	6.3	163.0755	$C_{10}H_{10}O_2$	Phenolic acids	[M+H]$^+$	131, 103	Level 2[a]
43	Caffeic acid	7.3	181.0495	$C_9H_8O_4$	Phenolic acids	[M+H]$^+$	163, 145, 89	Level 1
44	Ferulic acid	13.3	195.0655	$C_{10}H_{10}O_4$	Phenolic acids	[M+H]$^+$	177, 145	Level 1
45	Coumaric acid	11.2	165.0546	$C_9H_{10}O_3$	Phenolic acids	[M+H]$^+$	147, 119	Level 1
46	3,5-Dimethoxycinnamic acid	5.1	209.0808	$C_{11}H_{12}O_4$	Phenolic acids	[M+H]$^+$	149	Level 2[a]
47	Sinapic acid	12.7	225.0759	$C_{11}H_{12}O_5$	Phenolic acids	[M+H]$^+$	207, 175	Level 1
48	6,7-Dihydroxycoumarin-6-*O*-glucoside	5.6	339.0720	$C_{15}H_{16}O_9$	Phenolic acids	[M-H]$^-$	177, 137	Level 2[a]
49	Umbelliferone	7.0	161.0231	C9H6O3	Phenolic acids	[M-H]$^-$	133	Level 2[a]
50	Apigenin	21.4	269.0457	$C_{15}H_{10}O_5$	Flavonoids	[M-H]$^-$	251, 241, 223	Level 1
51	Apigenin-5-*O*-neohesperidoside	16.7	577.1552	$C_{27}H_{30}O_{14}$	Flavonoids	[M-H]$^-$	431, 269, 251, 223	Level 2[a]
52	Apigenin-7-*O*-neohesperidoside	17.1	577.1552	$C_{27}H_{30}O_{14}$	Flavonoids	[M-H]$^-$	431, 269, 251, 223	Level 2[a]
53	Apigenin-7-*O*-glucoside	17.2	431.0973	$C_{21}H_{20}O_{10}$	Flavonoids	[M-H]$^-$	269, 251, 223	Level 1
54	Luteolin	20.5	285.0398	$C_{15}H_{10}O_6$	Flavonoids	[M-H]$^-$	217, 175, 151, 133	Level 1
55	Luteoloside	15.5	447.0936	$C_{21}H_{20}O_{11}$	Flavonoids	[M-H]$^-$	285, 217, 175, 151, 133	Level 1
56	Kaempferol-3-*O*-glucoside	16.6	447.0926	$C_{21}H_{20}O_{11}$	Flavonoids	[M-H]$^-$	285	Level 1
57	Kaempferol-7-*O*-rutinoside I	15.2	593.1510	$C_{27}H_{30}O_{15}$	Flavonoids	[M-H]$^-$	447, 285, 268, 239, 221	Level 2[c]
58	Kaempferol-7-O-rutinoside	15.8	593.1510	$C_{27}H_{30}O_{15}$	Flavonoids	[M-H]$^-$	447, 285, 268, 239, 221	Level 2[b]
59	Kaempferol-7-O-rutinoside II	16.4	593.1510	$C_{27}H_{30}O_{15}$	Flavonoids	[M-H]$^-$	447, 285, 268, 239, 221	Level 2[b]
60	Velutin	21.0	313.0720	$C_{17}H_{14}O_6$	Flavonoids	[M-H]$^-$	301, 300, 271	Level 2[a]
61	Quercetin-3-*O*-vicianoside	14.3	595.1315	$C_{26}H_{28}O_{16}$	Flavonoids	[M-H]$^-$	300	Level 2[b]
62	Quercetin-7-*O*-rutinoside I	14.5	609.1461	$C_{27}H_{30}O_{16}$	Flavonoids	[M-H]$^-$	301, 271, 255, 178	Level 2[a]
63	Quercetin-7-*O*-rutinoside	14.9	609.1461	$C_{27}H_{30}O_{16}$	Flavonoids	[M-H]$^-$	301, 271, 255, 178	Level 2[a]
64	Quercetin-*O*-diglucoside	9.6	625.1417	$C_{27}H_{30}O_{17}$	Flavonoids	[M-H]$^-$	463, 301, 271	Level 2[a]
65	Isoquercitrin	15.3	463.0886	$C_{21}H_{20}O_{12}$	Flavonoids	[M-H]$^-$	301,179, 151	Level 1
66	Isorhamnetin-3-*O*-glucoside	17.2	477.1046	$C_{22}H_{22}O_{12}$	Flavonoids	[M-H]$^-$	315, 300, 271, 243, 179, 151	Level 1
67	Isorhamnetin-7-*O*-rutinoside I	16.4	623.1614	$C_{28}H_{32}O_{16}$	Flavonoids	[M-H]$^-$	315, 299, 271, 243	Level 2[c]

68	Isorhamnetin-7-*O*-rutinoside	16.7	623.1607	$C_{28}H_{32}O_{16}$	Flavonoids	$[M-H]^-$	315, 299, 271, 243, 178, 151	Level 2[b]
69	Eriodictyol-7-*O*-glucoside	14.7	449.1080	$C_{21}H_{22}O_{11}$	Flavonoids	$[M-H]^-$	415, 315, 299, 298, 163	Level 2[c]
70	Eupatilin	21.1	343.0824	$C_{18}H_{16}O_7$	Flavonoids	$[M-H]^-$	328	Level 2[a]
71	Acacetin	22.8	283.0611	$C_{16}H_{12}O_5$	Flavonoids	$[M-H]^-$	268	Level 2[a]
72	Chryseriol	21.4	299.0562	$C_{16}H_{12}O_6$	Flavonoids	$[M-H]^-$	256, 284, 285	Level 2[a]
73	Riboflavin	11.2	375.1308	$C_{17}H_{20}N_4O_6$	Flavonoids	$[M-H]^-$	255, 193, 119	Level 2[a]
74	6-Geranylnaringenin	15.7	407.1872	$C_{25}H_{28}O_5$	Flavonoids	$[M-H]^-$	287	Level 2[a]
75	Irisxanthone	20.2	435.0940	$C_{20}H_{20}O_{11}$	Flavonoids	$[M-H]^-$	315	Level 2[a]
76	Diosmin	17.6	607.1679	$C_{28}H_{32}O_{15}$	Flavonoids	$[M-H]^-$	300, 299, 284	Level 2[a]
77	Rutin	14.9	609.1464	$C_{27}H_{30}O_{16}$	Flavonoids	$[M-H]^-$	300, 271, 255, 178, 151	Level 1
78	Kaempferol-*O*-acetylglucoside	17.5	489.1027	$C_{23}H_{22}O_{12}$	Flavonoids	$[M-H]^-$	285, 255, 227, 153	Level 2[c]
79	Quercetin-*O*-acetylglucoside	16.1	505.0977	$C_{23}H_{22}O_{13}$	Flavonoids	$[M-H]^-$	300, 271, 255, 234, 179, 151	Level 2[c]
80	Quercetin-*O*-malonylglucoside	16.1	549.0875	$C_{24}H_{22}O_{15}$	Flavonoids	$[M-H]^-$	300, 271, 255, 234, 179, 151	Level 2[c]
81	Kaempferol	21.6	285.0401	$C_{15}H_{10}O_6$	Flavonoids	$[M+H]^-$	268, 239, 221	Level 1
82	4',7-Dimethoxy-3-hydroxyflavone	24.4	299.0912	$C_{17}H_{14}O_5$	Flavonoids	$[M+H]^+$	284, 256	Level 2[a]
83	Tricetin	14.9	303.0496	$C_{15}H_{10}O_7$	Flavonoids	$[M+H]^+$	153	Level 2[a]
84	5-Hydroxyl-3,4,7-trimethoxy flavone	23.9	329.1014	$C_{18}H_{16}O_6$	Flavonoids	$[M+H]^+$	314, 313	Level 2[a]
85	Corymbosin	24.1	359.1120	$C_{19}H_{18}O_7$	Flavonoids	$[M+H]^+$	329	Level 2[a]
86	Diosmetin-5-*O*-glucoside	17.8	463.1235	$C_{22}H_{22}O_{11}$	Flavonoids	$[M+H]^+$	301, 286, 258, 153	Level 2[a]
87	Diosmetin-7-*O*-glucoside	20.9	463.1239	$C_{22}H_{22}O_{11}$	Flavonoids	$[M+H]^+$	301, 286, 258, 153	Level 2[a]
88	Flavoyadorinin B	21.0	477.1391	$C_{23}H_{24}O_{11}$	Flavonoids	$[M+H]^+$	315, 300	Level 2[a]
89	Arbutoside I	6.8	697.2195	$C_{28}H_{42}O_{20}$	Flavonoids	$[M-H]^-$	535, 373, 355, 341	Level 2[a]
90	Arbutoside	9.3	697.2195	$C_{28}H_{42}O_{20}$	Flavonoids	$[M-H]^-$	535, 373, 355, 341	Level 2[a]
91	Arbutoside II	10.0	697.2195	$C_{28}H_{42}O_{20}$	Flavonoids	$[M-H]^-$	535, 373, 355, 341	Level 2[a]
92	Deoxyloganic acid	13.7	359.1350	$C_{16}H_{24}O_9$	Iridoids	$[M-H]^-$	197, 153, 135, 109	Level 2[a]
93	Secologanic acid	8.2	373.1147	$C_{16}H_{22}O_{10}$	Iridoids	$[M-H]^-$	193, 167, 149, 119, 97	Level 1
94	Secologanic acid I	7.9	373.1147	$C_{16}H_{22}O_{10}$	Iridoids	$[M-H]^-$	193, 167, 149, 119, 97	Level 2[c]
95	Geniposidic acid	3.5	373.1136	$C_{16}H_{22}O_{10}$	Iridoids	$[M-H]^-$	211, 167, 149	Level 2[c]
96	Swertiamarin	5.5	373.1139	$C_{16}H_{22}O_{10}$	Iridoids	$[M-H]^-$	211, 167, 149, 123	Level 1
97	8-epi-Loganic acid	3.8	375.1297	$C_{16}H_{24}O_{10}$	Iridoids	$[M-H]^-$	213, 169, 151, 125	Level 2[c]
98	Loganic acid	6.1	375.1297	$C_{16}H_{24}O_{10}$	Iridoids	$[M-H]^-$	213, 169, 151, 125	Level 1
99	7-epi-Loganic acid	4.8	375.1297	$C_{16}H_{24}O_{10}$	Iridoids	$[M-H]^-$	213, 169, 151, 125	Level 2[c]
100	Demethylsecologanol	7.0	375.1294	$C_{16}H_{24}O_{10}$	Iridoids	$[M-H]^-$	195, 169, 151	Level 2[c]
101	Secologanoside I	2.6	389.1087	$C_{16}H_{22}O_{11}$	Iridoids	$[M-H]^-$	345, 209, 183, 165	Level 2[b]
102	Secologanoside II	3.5	389.1087	$C_{16}H_{22}O_{11}$	Iridoids	$[M-H]^-$	345, 209, 183, 165	Level 2[b]

103	Secologanoside	7.8	389.1087	$C_{16}H_{22}O_{11}$	Iridoids	$[M-H]^-$	345, 209, 183, 165	Level 1
104	Demethyl-Morroniside	2.0	391.1242	$C_{16}H_{24}O_{11}$	Iridoids	$[M-H]^-$	229, 211, 193, 123	Level 2[b]
105	Demethyl-Morroniside I	2.2	391.1242	$C_{16}H_{24}O_{11}$	Iridoids	$[M-H]^-$	229, 211, 193, 123	Level 2[b]
106	Secoxyloganin	12.5	403.1244	$C_{17}H_{24}O_{11}$	Iridoids	$[M-H]^-$	223, 179, 165, 121, 119	Level 1
107	Secoxyloganin I	6.3	403.1244	$C_{17}H_{24}O_{11}$	Iridoids	$[M-H]^-$	223, 209, 191, 149, 101	Level 2[c]
108	Secoxyloganin II	8.6	403.1244	$C_{17}H_{24}O_{11}$	Iridoids	$[M-H]^-$	223, 209, 191, 149, 101	Level 2[c]
109	Morroniside	7.0	405.1403	$C_{17}H_{26}O_{11}$	Iridoids	$[M-H]^-$	373, 243, 155, 141	Level 1
110	Morroniside I	12.4	405.1403	$C_{17}H_{26}O_{11}$	Iridoids	$[M-H]^-$	373, 243, 155, 141	Level 2[c]
111	Dimethylsecologanoside	13.7	417.1405	$C_{18}H_{26}O_{11}$	Iridoids	$[M-H]^-$	255, 237, 185, 163, 155	Level 2[a]
112	7-O-Methyl morroniside	12.0	419.1548	$C_{18}H_{28}O_{11}$	Iridoids	$[M-H]^-$	239, 213, 155, 119	Level 1
113	Genameside A I	4.0	421.1345	$C_{17}H_{26}O_{12}$	Iridoids	$[M-H]^-$	241, 197	Level 2[c]
114	Genameside A	4.5	421.1345	$C_{17}H_{26}O_{12}$	Iridoids	$[M-H]^-$	241, 197	Level 2[b]
115	Genameside A II	5.2	421.1345	$C_{17}H_{26}O_{12}$	Iridoids	$[M-H]^-$	241, 197	Level 2[b]
116	Geniposide	10.5	433.1351	$C_{18}H_{26}O_{12}$	Iridoids	$[M+HCOO]^-$	225, 207, 123	Level 1
117	Epi-vogeloside	12.6	433.1353	$C_{18}H_{26}O_{12}$	Iridoids	$[M+HCOO]^-$	387, 225, 155	Level 1
118	Dehydromorroniside	13.0	433.1361	$C_{18}H_{26}O_{12}$	Iridoids	$[M+HCOO]^-$	387, 225, 207, 155, 123	Level 2[b]
119	Loganin I	10.2	435.1500	$C_{18}H_{28}O_{12}$	Iridoids	$[M+HCOO]^-$	389, 227, 209, 127,	Level 2[b]
120	Loganin	11.2	435.1500	$C_{18}H_{28}O_{12}$	Iridoids	$[M+HCOO]^-$	389, 227, 209, 127,	Level 1
121	Loganin II	12.5	435.1500	$C_{18}H_{28}O_{12}$	Iridoids	$[M+HCOO]^-$	389, 227, 209, 127,	Level 2[b]
122	Secologanside-7-methyl ester	12.5	449.1296	$C_{18}H_{26}O_{13}$	Iridoids	$[M+HCOO]^-$	403, 241, 223, 179	Level 2[a]
123	L-phenylalaninosecologanin B	20.4	504.1878	$C_{25}H_{31}NO_{10}$	Iridoids	$[M-H]^-$	342, 324, 272, 228	Level 2[a]
124	Demethyl-strychoside A	14.3	729.2254	$C_{32}H_{42}O_{19}$	Iridoids	$[M-H]^-$	505, 453, 409	Level 2[a]
125	Demethyl-strychoside A I	16.6	729.2254	$C_{32}H_{42}O_{19}$	Iridoids	$[M-H]^-$	497, 453, 409	Level 2[a]
126	Strychoside A	15.6	743.2402	$C_{33}H_{44}O_{19}$	Iridoids	$[M-H]^-$	581, 563, 511, 467	Level 2[b]
127	Strychoside A I	16.9	743.2402	$C_{33}H_{44}O_{19}$	Iridoids	$[M-H]^-$	581, 563, 511, 467	Level 2[b]
128	(E)-Aldosecologanin	17.4	757.2575	$C_{34}H_{46}O_{19}$	Iridoids	$[M-H]^-$	595, 525, 493	Level 1
129	(Z)-Aldosecologanin	18.1	757.2575	$C_{34}H_{46}O_{19}$	Iridoids	$[M-H]^-$	595, 525, 493	Level 1
130	Genipin	5.1	227.0915	$C_{11}H_{14}O_5$	Iridoids	$[M+H]^+$	149, 103	Level 2[a]
131	Gentiopicroside	5.6	357.1183	$C_{16}H_{20}O_9$	Iridoids	$[M-H]^-$	195, 177, 121	Level 2[a]
132	Sweroside	10.7	403.1252	$C_{17}H_{24}O_{11}$	Iridoids	$[M+HCOO]^-$	357, 195, 125	Level 1
133	Sweroside I	4.0	403.1252	$C_{17}H_{24}O_{11}$	Iridoids	$[M+HCOO]^-$	357, 195, 125	Level 2[c]
134	Sweroside II	4.9	403.1252	$C_{17}H_{24}O_{11}$	Iridoids	$[M+HCOO]^-$	357, 195, 125	Level 2[c]
135	Roseoside	10.9	387.2014	$C_{19}H_{30}O_8$	Iridoids	$[M+H]^+$	225, 207, 123	Level 2[a]
136	Roseoside I	11.4	387.2015	$C_{19}H_{30}O_8$	Iridoids	$[M+H]^+$	225, 207, 123	Level 2[a]
137	Vogeloside I	12.6	387.1289	$C_{17}H_{24}O_{10}$	Iridoids	$[M-H]^-$	225, 207, 155	Level 2[c]

138	Vogeloside	13.1	387.1289	$C_{17}H_{24}O_{10}$	Iridoids	$[M-H]^-$	225, 207, 155	Level 1
139	7-*O*-Ethylsweroside	16.5	403.1591	$C_{18}H_{26}O_{10}$	Iridoids	$[M+H]^+$	241, 209, 177	Level 2^a
140	Lonijaposide K	12.3	494.1657	$C_{23}H_{27}NO_{11}$	Iridoids	$[M+H]^+$	262	Level 2^a
141	Lonijaposide O	19.1	508.1817	$C_{24}H_{29}NO_{11}$	Iridoids	$[M+H]^+$	346, 328, 276	Level 2^a
142	Loganic acid-*O*-pentoside	9.5	509.1868	$C_{21}H_{32}O_{14}$	Iridoids	$[M+H]^+$	377	Level 2^a
143	Lonijaposide M	6.0	522.1968	$C_{25}H_{31}NO_{11}$	Iridoids	$[M+H]^+$	360, 290, 246	Level 2^a
144	Lonijaposide N	10.2	536.2124	$C_{26}H_{33}NO_{11}$	Iridoids	$[M+H]^+$	372	Level 2^a
145	Dimethyl lonijaposide C	11.7	552.2076	$C_{26}H_{33}NO_{12}$	Iridoids	$[M+H]^+$	372	Level 2^a
146	Hydro-dimethyl lonijaposide C	10.9	554.2228	$C_{26}H_{35}NO_{12}$	Iridoids	$[M+H]^+$	392, 374	Level 2^a
147	5a-Carboxystrictosidine I	14.7	575.2234	$C_{28}H_{34}N_2O_{11}$	Iridoids	$[M+H]^+$	413, 395, 343	Level 2^a
148	5a-Carboxystrictosidine	15.9	575.2228	$C_{28}H_{34}N_2O_{11}$	Iridoids	$[M+H]^+$	413, 395, 343	Level 2^a
149	5a-Carboxystrictosidine II	18.0	575.2229	$C_{28}H_{34}N_2O_{11}$	Iridoids	$[M+H]^+$	413, 395, 343	Level 2^a
150	Lonijaposide H	9.7	580.2015	$C_{27}H_{33}NO_{13}$	Iridoids	$[M+H]^+$	418, 348, 262, 230	Level 2^a
151	Lonijaposide H I	12.4	580.2022	$C_{27}H_{33}NO_{13}$	Iridoids	$[M+H]^+$	418, 348, 262, 230	Level 2^a
152	Lonijaposide H II	13.0	580.2029	$C_{27}H_{33}NO_{13}$	Iridoids	$[M+H]^+$	418, 348, 262, 230	Level 2^a
153	Methyl-lonijaposide T I	15.3	608.2331	$C_{29}H_{37}NO_{13}$	Iridoids	$[M+H]^+$	446, 376	Level 2^a
154	Methyl-lonijaposide T	15.9	608.2331	$C_{29}H_{37}NO_{13}$	Iridoids	$[M+H]^+$	446, 376	Level 2^a
155	Hydro-lonijaposide N	14.0	538.2283	$C_{26}H_{35}NO_{11}$	Iridoids	$[M+H]^+$	376, 358, 326, 298	Level 2^a
156	Lonijaposide T	13.1	594.2190	$C_{28}H_{35}NO_{13}$	Iridoids	$[M+H]^+$	362	Level 2^a
157	Sweroside aglycone	7.0	197.0809	$C_{10}H_{12}O_4$	Iridoids	$[M+H]^+$	179, 151, 127	Level 2^a

Note: Level 1: Confirmed by reference standards (RT and MS/MS match).

Level 2^a : Identification based on database and/or literature comparison combined with MS/MS data.

Level 2^b: Identification supported by molecular networking.

Level 2^c: Identification based on fragmentation pattern analysis.

Table S4 Primary metabolites identified in the processed LJF samples.

NO.	Name	t_R/min	*m/z*	Formula	Classification	Mode	Identification
1	Pyroglutamic acid	1.1	128.0348	$C_5H_7NO_3$	Amino acids	$[M-H]^-$	Level 2^a
2	L-isoleucine	1.3	130.0869	$C_6H_{13}NO_2$	Amino acids	$[M-H]^-$	Level 1
3	Phenylalanine	2.2	166.0861	$C_9H_{11}NO_2$	Amino acids	$[M+H]^+$	Level 1
4	Tyrosine	1.2	182.0811	$C_9H_{11}NO_3$	Amino acids	$[M+H]^+$	Level 1
5	N-Acetylleucine	5.7	196.0969	$C_8H_{15}NO_3$	Amino acids	$[M+H]^+$	Level 2^a
6	Tryptophan	4.4	205.0974	$C_{11}H_{12}N_2O_2$	Amino acids	$[M+H]^+$	Level 1
7	Glutamine	1.2	147.0706	$C_5H_{10}N_2O_3$	Amino acids	$[M+H]^+$	Level 1
8	Serine	1.6	106.0500	$C_3H_7NO_3$	Amino acids	$[M+H]^+$	Level 1
9	Leucine	1.2	132.1020	$C_6H_{13}NO_2$	Amino acids	$[M+H]^+$	Level 1
10	Proline	0.8	116.0702	$C_5H_9NO_2$	Amino acids	$[M+H]^+$	Level 1
11	Glutamic acid	0.8	148.0611	$C_5H_9NO_4$	Amino acids	$[M+H]^+$	Level 1
12	Arginine	0.7	175.1193	$C_6H_{14}N_4O_2$	Amino acids	$[M+H]^+$	Level 1
13	Aspartic acid	0.8	134.0449	$C_4H_7NO_4$	Amino acids	$[M+H]^+$	Level 1
14	N-Fructosyl isoleucine	1.3	294.1548	$C_{12}H_{23}NO_7$	Amino acids	$[M+H]^+$	Level 2^a

15	N-Fructosyl phenylalanine	2.2	328.1389	$C_{15}H_{21}NO_7$	Amino acids	$[M+H]^+$	Level 2[a]
16	N-Fructosyl tyrosine	1.1	344.1341	$C_{15}H_{21}NO_8$	Amino acids	$[M+H]^+$	Level 2[a]
17	FA 12:1+1O	21.8	213.1495	$C_{12}H_{22}O_3$	Fatty acids	$[M-H]^-$	Level 2[a]
18	Undecanedioic acid	21.3	215.1285	$C_{11}H_{20}O_4$	Fatty acids	$[M-H]^-$	Level 2[a]
19	Omega-hydroxydodecanoic acid	22.2	215.1664	$C_{12}H_{24}O_3$	Fatty acids	$[M-H]^-$	Level 2[a]
20	FA 13:3+1O	22.7	223.1338	$C_{13}H_{20}O_3$	Fatty acids	$[M-H]^-$	Level 2[a]
21	FA 15:0	26.8	241.2171	$C_{15}H_{30}O_2$	Fatty acids	$[M-H]^-$	Level 2[a]
22	Tridecanedioic acid	22.5	243.1603	$C_{13}H_{24}O_4$	Fatty acids	$[M-H]^-$	Level 2[a]
23	FA 16:1	26.5	253.2170	$C_{16}H_{30}O_2$	Fatty acids	$[M-H]^-$	Level 1
24	FA 16:0	27.2	255.2329	$C_{16}H_{32}O_2$	Fatty acids	$[M-H]^-$	Level 1
25	FA 17:1	27.0	267.2329	$C_{17}H_{32}O_2$	Fatty acids	$[M-H]^-$	Level 2[a]
26	FA 17:0	27.5	269.2485	$C_{17}H_{34}O_2$	Fatty acids	$[M-H]^-$	Level 2[a]
27	FA 17:0	27.7	269.2488	$C_{17}H_{34}O_2$	Fatty acids	$[M-H]^-$	Level 2[a]
28	FA 18:3	26.1	277.2170	$C_{18}H_{30}O_2$	Fatty acids	$[M-H]^-$	Level 2[a]
29	FA 18:3	24.5	277.2172	$C_{18}H_{30}O_2$	Fatty acids	$[M-H]^-$	Level 2[a]
30	FA 18:2	26.6	279.2327	$C_{18}H_{32}O_2$	Fatty acids	$[M-H]^-$	Level 2[a]
31	FA 18:1	27.3	281.2487	$C_{18}H_{34}O_2$	Fatty acids	$[M-H]^-$	Level 2[a]
32	FA 18:0	28.3	283.2636	$C_{18}H_{36}O_2$	Fatty acids	$[M-H]^-$	Level 2[a]
33	FA 18:4+1O	24.4	291.1964	$C_{18}H_{28}O_3$	Fatty acids	$[M-H]^-$	Level 2[a]
34	FA 18:3+1O	24.0	293.2124	$C_{18}H_{30}O_3$	Fatty acids	$[M-H]^-$	Level 2[a]
35	FA 19:0	29.1	297.2802	$C_{19}H_{38}O_2$	Fatty acids	$[M-H]^-$	Level 2[a]
36	FA 18:5+2O	22.9	305.1754	$C_{18}H_{26}O_4$	Fatty acids	$[M-H]^-$	Level 2[a]
37	FA 18:4+2O	22.5	307.1916	$C_{18}H_{28}O_4$	Fatty acids	$[M-H]^-$	Level 2[a]
38	FA 20:2	27.5	307.2643	$C_{20}H_{36}O_2$	Fatty acids	$[M-H]^-$	Level 2[a]
39	FA 18:3+2O	23.3	309.2069	$C_{18}H_{30}O_4$	Fatty acids	$[M-H]^-$	Level 2[a]
40	FA 20:1	28.4	309.2800	$C_{20}H_{38}O_2$	Fatty acids	$[M-H]^-$	Level 2[a]
41	FA 18:2+2O	23.6	311.2231	$C_{18}H_{32}O_4$	Fatty acids	$[M-H]^-$	Level 2[a]
42	FA 20:0	29.9	311.2946	$C_{20}H_{40}O_2$	Fatty acids	$[M-H]^-$	Level 2[a]
43	FA 18:1+2O	23.5	313.2386	$C_{18}H_{34}O_4$	Fatty acids	$[M-H]^-$	Level 2[a]
44	FA 18:3+3O	22.2	325.2019	$C_{18}H_{30}O_5$	Fatty acids	$[M-H]^-$	Level 2[a]
45	FA 21:0	31.0	325.3109	$C_{21}H_{42}O_2$	Fatty acids	$[M-H]^-$	Level 2[a]
46	FA 18:2+3O	21.8	327.2175	$C_{18}H_{32}O_5$	Fatty acids	$[M-H]^-$	Level 2[a]
47	FA 18:2+3O	22.1	327.2177	$C_{18}H_{32}O_5$	Fatty acids	$[M-H]^-$	Level 2[a]
48	FA 18:1+3O	22.1	329.2335	$C_{18}H_{34}O_5$	Fatty acids	$[M-H]^-$	Level 2[a]
49	FA 18:2+4O	19.8	343.2130	$C_{18}H_{32}O_6$	Fatty acids	$[M-H]^-$	Level 2[a]
50	FA 23:0	28.7	353.3428	$C_{23}H_{46}O_2$	Fatty acids	$[M-H]^-$	Level 2[a]
51	FA 24:0	30.9	367.3578	$C_{24}H_{48}O_2$	Fatty acids	$[M-H]^-$	Level 2[a]
52	FA 26:6	25.0	383.2925	$C_{26}H_{40}O_2$	Fatty acids	$[M-H]^-$	Level 2[a]
53	FA 26:5	27.2	385.3115	$C_{26}H_{42}O_2$	Fatty acids	$[M-H]^-$	Level 2[a]
54	FA 26:5	29.2	385.3115	$C_{26}H_{42}O_2$	Fatty acids	$[M-H]^-$	Level 2[a]
55	FA 26:0	30.3	425.3636	$C_{26}H_{50}O_4$	Fatty acids	$[M-H]^-$	Level 2[a]

56	FA18:4	24.0	275.1995	$C_{18}H_{28}O_2$	Fatty acids	$[M-H]^-$	Level 2[a]
57	FA 16:1	26.5	253.2170	$C_{16}H_{30}O_2$	Fatty acids	$[M-H]^-$	Level 2[a]
58	FA 18:1+3O	21.7	329.2332	$C_{18}H_{34}O_5$	Fatty acids	$[M-H]^-$	Level 2[a]
59	Lyso PE 16:0	24.8	496.3397	$C_{24}H_{50}NO_7P$	Lipids	$[M+H]^+$	Level 1
60	Lyso PE 18:2	24.2	520.3387	$C_{26}H_{50}NO_7P$	Lipids	$[M+H]^+$	Level 1
61	Lyso PE 18:3	23.5	518.3229	$C_{26}H_{48}NO_7P$	Lipids	$[M+H]^+$	Level 1
62	Lyso PE 18:2	23.8	478.2917	$C_{23}H_{44}NO_7P$	Lipids	$[M+H]^+$	Level 1
63	Lyso PE 18:3	23.3	476.2763	$C_{23}H_{42}NO_7P$	Lipids	$[M+H]^+$	Level 1
64	Guanine	1.2	150.0418	$C_5H_5N_5O$	Nucleotides	$[M-H]^-$	Level 1
65	Guanosine	1.2	282.0845	$C_{10}H_{13}N_5O_5$	Nucleotides	$[M-H]^-$	Level 1
66	N2,N2-Dimethylguanosine	3.0	310.1152	$C_{12}H_{17}N_5O_5$	Nucleotides	$[M-H]^-$	Level 2[a]
67	Adenine	0.9	136.0619	$C_5H_5N_5$	Nucleotides	$[M+H]^+$	Level 1
68	5-Formyl-deoxyuridine	23.5	256.2636	$C_{23}H_{24}O_{11}$	Nucleotides	$[M+H]^+$	Level 2[a]
69	N6-Me-Adenosine	1.9	282.1197	$C_{11}H_{15}N_5O_4$	Nucleotides	$[M+H]^+$	Level 2[a]
70	Methylthioadenosine	4.8	298.0971	$C_{11}H_{15}N_5O_3S$	Nucleotides	$[M+H]^+$	Level 2[a]
71	3'-*O*-Methylguanosine	1.1	298.1158	$C_{11}H_{15}N_5O_5$	Nucleotides	$[M+H]^+$	Level 1
72	1-Pyridylhydroxybutyl-deoxyinosine	1.7	402.1755	$C_{19}H_{23}N_5O_5$	Nucleotides	$[M+H]^+$	Level 2[a]
73	Uracil	1.1	113.0346	$C_4H_4N_2O_2$	Nucleotides	$[M+H]^+$	Level 2[a]
74	Serylleucine	2.6	219.1339	$C_9H_{18}N_2O_4$	Dipeptides	$[M+H]^+$	Level 2[a]
75	Gamma-Glutamylleucine	4.9	261.1444	$C_{11}H_{20}N_2O_5$	Dipeptides	$[M+H]^+$	Level 2[a]
76	Ala-Phe	1.2	237.1233	$C_{12}H_{16}N_2O_3$	Dipeptides	$[M+H]^+$	Level 2[a]
77	Ile-Ile	8.9	245.1859	$C_{12}H_{24}N_2O_3$	Dipeptides	$[M+H]^+$	Level 2[a]
78	Asn-Leu	2.7	246.1446	$C_{10}H_{19}N_3O_4$	Dipeptides	$[M+H]^+$	Level 2[a]
79	Malic acid	0.9	133.0135	$C_4H_6O_5$	Organic acids	$[M-H]^-$	Level 1
80	Alpha-hydroxyglutaric acid	0.9	147.0292	$C_5H_8O_5$	Organic acids	$[M-H]^-$	Level 2[a]
81	3-Hydroxy-3-methylglutaric acid	1.4	161.0451	$C_6H_{10}O_5$	Organic acids	$[M-H]^-$	Level 2[a]
82	2-Isopropylmalic acid	5.5	175.0608	$C_7H_{12}O_5$	Organic acids	$[M-H]^-$	Level 2[a]
83	3-Phenyllactic acid	12.5	167.0704	$C_9H_{10}O_3$	Organic acids	$[M+H]^+$	Level 2[a]
84	3,4-Dimethoxyphenylacetic acid	6.2	197.0808	$C_{10}H_{12}O_4$	Organic acids	$[M+H]^+$	Level 2[a]
85	Citric acid	1.2	191.0189	$C_6H_8O_7$	Organic acids	$[M-H]^-$	Level 1
86	Synapic acid	19.2	207.0650	$C_{11}H_{12}O_5$	Organic acids	$[M+H]^+$	Level 2[a]
87	Isocitric acid	0.9	191.0189	$C_6H_8O_7$	Organic acids	$[M-H]^-$	Level 1
88	Succinic acid	1.2	117.0185	$C_4H_6O_4$	Organic acids	$[M-H]^-$	Level 1
89	Fumaric acid	0.8	115.0029	$C_4H_4O_4$	Organic acids	$[M-H]^-$	Level 1
90	Rhizonic acid	4.9	197.0810	$C_{10}H_{12}O_4$	Organic acids	$[M+H]^+$	Level 2[a]
91	Folinic acid	4.9	474.1733	$C_{20}H_{23}N_7O_7$	Organic acids	$[M-H]^-$	Level 2[a]
92	Mannose	0.7	179.0553	$C_6H_{12}O_6$	Sugars	$[M-H]^-$	Level 1
93	Glucose	0.8	179.0564	$C_6H_{12}O_6$	Sugars	$[M-H]^-$	Level 1
94	Sucrose	0.8	341.1093	$C_{12}H_{22}O_{11}$	Sugars	$[M-H]^-$	Level 1
95	Pantothenic acid	2.6	218.1034	$C_9H_{17}NO_5$	Vitamins	[M-H]–	Level 2[a]

96	Nicotinic acid	0.8	124.0394	$C_6H_5NO_2$	Vitamins	$[M+H]^+$	Level 1
97	Abscisic acid	20.0	263.1277	$C_{15}H_{20}O_4$	Others	$[M-H]^-$	Level 2[a]
98	N-Fructosyl pyroglutamate	1.1	290.0888	$C_{11}H_{17}NO_8$	Others	$[M-H]^-$	Level 2[a]
99	Pinoresinol-4-*O*-glucoside	16.4	519.1873	$C_{26}H_{32}O_{11}$	Others	$[M-H]^-$	Level 2[a]
100	3-Hydroxy-2-methyl-4-pyrone	10.7	127.0391	$C_6H_6O_3$	Others	$[M+H]^+$	Level 2[a]
101	p-Coumaraldehyde	7.0	149.0597	$C_9H_8O_2$	Others	$[M+H]^+$	Level 2[a]
102	Feruloylputrescine	8.1	265.1547	$C_{14}H_{20}N_2O_3$	Others	$[M+H]^+$	Level 2[a]
103	Syringin	8.0	390.1756	$C_{17}H_{24}O_9$	Others	$[M+NH4]^+$	Level 2[a]

Note: Level 1: Confirmed by reference standards (RT and MS/MS match).

Level 2[a] : Identification based on database and/or literature comparison combined with MS/MS data.

Table S5. Contents of 22 compounds in decocted samples (mean ± SD, *n* = 3).

NO.	Name	0 min	15min	30min	1h	2h	3h
1	3-*O*-*p*-Coumaroylquinic acid	7.30 ± 0.22	10.00 ± 0.49	13.29 ± 1.95	23.30 ± 2.66	30.76 ± 1.35	38.80 ± 0.75
2	5-*O*-*p*-Coumaroylquinic acid	212.36 ± 172.17	265.01 ± 9.48	229.34 ± 12.42	193.85 ± 1.07	169.52 ± 11.14	148.99 ± 9.83
3	3-*O*-Caffeoylquinic acid	2008.47 ± 509.16	3843.46 ± 178.38	5796.45 ± 1009.45	8747.61 ± 67.90	11902.05 ± 365.07	13576.29 ± 278.01
4	5-*O*-Caffeoylquinic acid	55754.68 ± 4947.79	51518.33 ± 1699.63	46650.83 ± 3408.45	37894.00 ± 4515.84	35628.47 ± 554.90	32783.05 ± 1188.07
5	4-*O*-Caffeoylquinic acid	2611.33 ± 463.34	4538.64 ± 164.83	5490.80 ± 538.14	6044.01 ± 240.84	6943.37 ± 135.46	7329.63 ± 211.69
6	3-*O*-Feruloylquinic acid	2.48 ± 0.03	4.72 ± 0.10	7.86 ± 1.43	14.60 ± 1.34	19.98 ± 0.40	25.10 ± 0.10
7	5-*O*-Feruloylquinic acid	560.34 ± 66.49	553.63 ± 18.04	515.27 ± 41.41	407.09 ± 43.33	402.36 ± 20.32	388.95 ± 8.17
8	3,4-*O*-Di-caffeoylquinic acid	1410.27 ± 985.38	5813.43 ± 908.73	7655.36 ± 1532.85	5954.66 ± 1790.57	6541.75 ± 400.39	5368.61 ± 49.85
9	3,5-*O*-Di-caffeoylquinic acid	30656.65 ± 7570.84	32467.48 ± 4250.58	26129.55 ± 6389.18	13945.35 ± 4519.95	12157.56 ± 732.53	9535.57 ± 78.14
10	4,5-*O*-Di-caffeoylquinic acid	2043.42 ± 1532.91	7887.57 ± 1340.62	9693.86 ± 2107.24	6674.61 ± 2435.48	7019.22 ± 423.46	5528.85 ± 33.94
11	Luteoloside	538.96 ± 131.00	668.49 ± 60.24	630.19 ± 100.33	428.46 ± 103.32	391.19 ± 15.65	357.81 ± 19.36
12	Isoquercitrin	187.09 ± 73.57	266.37 ± 47.17	288.41 ± 59.57	173.53 ± 61.05	167.76 ± 13.10	127.43 ± 2.71
13	Kaempferol-3-*O*-glucoside	71.87 ± 8.17	96.31 ± 1.99	98.29 ± 7.16	78.66 ± 10.73	82.17 ± 0.55	82.43 ± 2.28
14	Loganic acid	959.39 ± 56.93	925.49 ± 30.06	818.75 ± 52.48	674.99 ± 8.98	641.64 ± 69.41	707.89 ± 10.40
15	Secologanoside	2592.84 ± 105.67	2710.22 ± 124.37	2238.25 ± 110.38	2536.82 ± 76.57	3307.08 ± 321.58	3376.19 ± 95.53
16	Morroniside	4176.32 ± 206.49	3922.20 ± 90.42	3574.90 ± 143.46	3236.52 ± 19.44	3403.58 ± 221.66	3589.82 ± 8.43
17	Epi-vogeloside	135.40 ± 7.88	131.95 ± 14.28	121.99 ± 17.80	112.98 ± 4.41	143.68 ± 3.95	156.80 ± 10.22
18	Loganin	2349.52 ± 271.77	2027.13 ± 75.15	2040.93 ± 90.25	1845.54 ± 123.20	1846.67 ± 176.07	1937.44 ± 43.98
19	(E)-Aldosecologanin	11891.47 ± 464.34	12179.29 ± 201.88	10327.43 ± 591.50	8986.57 ± 116.53	8667.19 ± 720.89	8453.42 ± 58.18
20	(Z)-Aldosecologanin	5471.09 ± 175.14	5287.78 ± 91.71	4500.66 ± 285.48	3706.88 ± 138.63	3317.09 ± 250.41	2975.06 ± 43.09
21	Sweroside	378.38 ± 16.31	438.71 ± 6.76	408.49 ± 23.64	460.80 ± 40.42	438.29 ± 184.69	563.40 ± 42.60
22	7-*O*-Methyl morroniside	100.95 ± 19.05	50.43 ± 15.75	79.75 ± 4.92	83.96 ± 11.12	135.47 ± 16.55	171.40 ± 15.67

Table S6. Contents of 22 compounds in macerated samples (mean ± SD, *n* = 3).

NO.	Name	1h	2h	3h	6h	12h	24h
1	3-*O*-*p*-Coumaroylquinic acid	4.81 ± 0.14	4.76 ± 0.10	5.02 ± 0.15	5.24 ± 0.59	5.92 ± 0.02	5.95 ± 0.57
2	5-*O*-*p*-Coumaroylquinic acid	224.55 ± 7.44	208.96 ± 9.53	224.79 ± 6.72	212.58 ± 31.32	225.37 ± 2.81	201.17 ± 14.19
3	3-*O*-Caffeoylquinic acid	4.96 ± 1.04	4.53 ± 0.43	5.47 ± 0.67	4.74 ± 0.30	5.59 ± 0.15	5.34 ± 0.74
4	5-*O*-Caffeoylquinic acid	91.68 ± 11.99	101.40 ± 5.30	106.41 ± 16.66	84.89 ± 2.36	78.30 ± 4.58	65.67 ± 15.51
5	4-*O*-Caffeoylquinic acid	4.48 ± 0.97	4.28 ± 0.45	4.30 ± 0.56	3.67 ± 0.24	3.47 ± 0.18	3.59 ± 0.60
6	3-*O*-Feruloylquinic acid	0.34 ± 0.06	0.38 ± 0.03	0.45 ± 0.03	0.46 ± 0.04	0.57 ± 0.02	0.64 ± 0.07
7	5-*O*-Feruloylquinic acid	48.04 ± 6.49	28.13 ± 7.25	41.70 ± 2.73	19.11 ± 3.64	60.13 ± 1.96	59.81 ± 4.63
8	3,4-*O*-Dicaffeoylquinic acid	8.08 ± 3.93	4.31 ± 0.19	3.44 ± 0.04	3.09 ± 0.24	2.86 ± 0.34	2.75 ± 0.20
9	3,5-*O*-Dicaffeoylquinic acid	40.69 ± 12.17	27.39 ± 1.55	24.24 ± 1.28	22.61 ± 0.59	21.92 ± 0.67	19.51 ± 0.84
10	4,5-*O*-Dicaffeoylquinic acid	9.05 ± 3.52	4.98 ± 0.42	4.30 ± 0.28	3.96 ± 0.10	3.67 ± 0.33	2.96 ± 0.52
11	Luteoloside	5.40 ± 2.41	2.50 ± 0.15	1.64 ± 0.15	1.28 ± 0.11	1.43 ± 0.26	0.85 ± 0.06
12	Isoquercitrin	0.85 ± 0.20	0.80 ± 0.03	0.81 ± 0.07	0.66 ± 0.07	0.89 ± 0.05	0.80 ± 0.01
13	Kaempferol-3-*O*-glucoside	2.67 ± 0.67	3.60 ± 0.98	4.41 ± 0.45	6.56 ± 3.19	8.09 ± 0.29	6.70 ± 0.63
14	Loganic acid	848.81 ± 15.41	816.38 ± 48.87	848.21 ± 22.01	878.74 ± 72.69	895.06 ± 18.63	872.01 ± 64.99
15	Secologanoside	2324.03 ± 12.96	2236.75 ± 118.56	2245.91 ± 24.67	2256.07 ± 133.86	2273.30 ± 53.88	2172.35 ± 211.89
16	Morroniside	3989.08 ± 58.79	3968.18 ± 132.10	4091.76 ± 50.95	3665.46 ± 204.54	3710.69 ± 101.05	3043.14 ± 107.14
17	Epi-vogeloside	216.81 ± 1.78	216.04 ± 10.91	227.87 ± 9.49	112.54 ± 9.87	110.71 ± 4.03	87.36 ± 17.09
18	Loganin	1901.15 ± 79.87	1947.36 ± 114.56	1932.40 ± 65.13	1772.63 ± 200.08	1702.75 ± 52.17	1517.13 ± 122.16
19	(E)-Aldosecologanin	8780.29 ± 357.39	9007.76 ± 574.91	9332.76 ± 346.17	8626.99 ± 588.70	8974.94 ± 239.09	8042.34 ± 853.99
20	(Z)-Aldosecologanin	4032.96 ± 133.32	4064.90 ± 258.46	4236.39 ± 86.24	3842.07 ± 295.54	4000.49 ± 62.85	3457.07 ± 285.62
21	Sweroside	259.96 ± 6.18	262.18 ± 9.58	267.89 ± 7.48	249.69 ± 40.67	286.27 ± 10.17	267.44 ± 6.88
22	7-*O*-Methyl morroniside	324.56 ± 13.11	335.20 ± 11.08	354.59 ± 10.49	99.65 ± 18.21	92.97 ± 19.07	92.91 ± 38.36

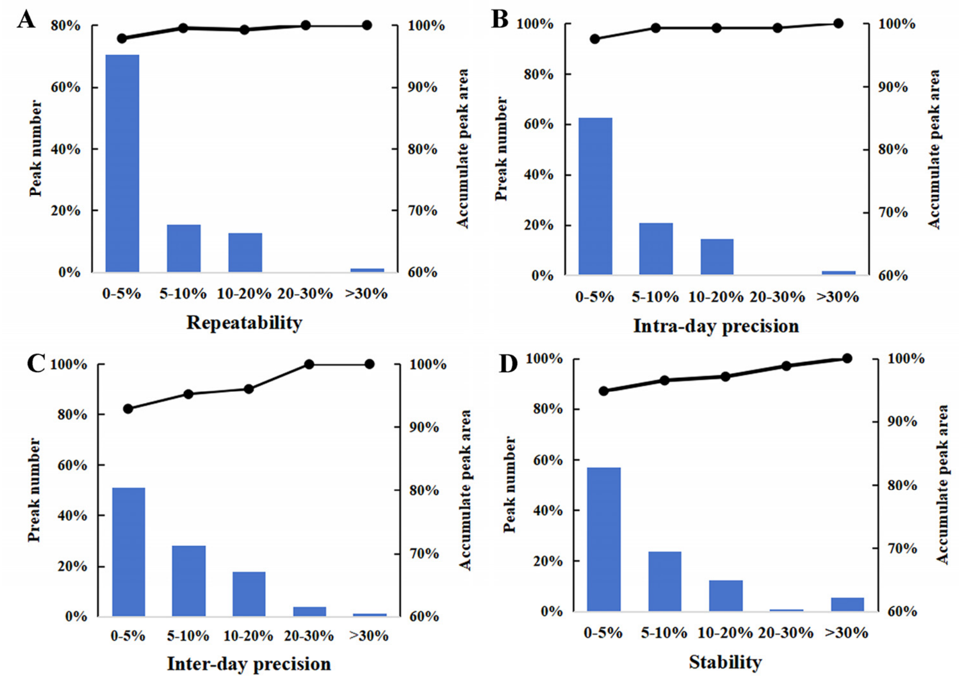

Figure S1. The peak number and accumulate peak area of repeatability (A) , precision (B, C) , stability (D) in different RSD ranges.

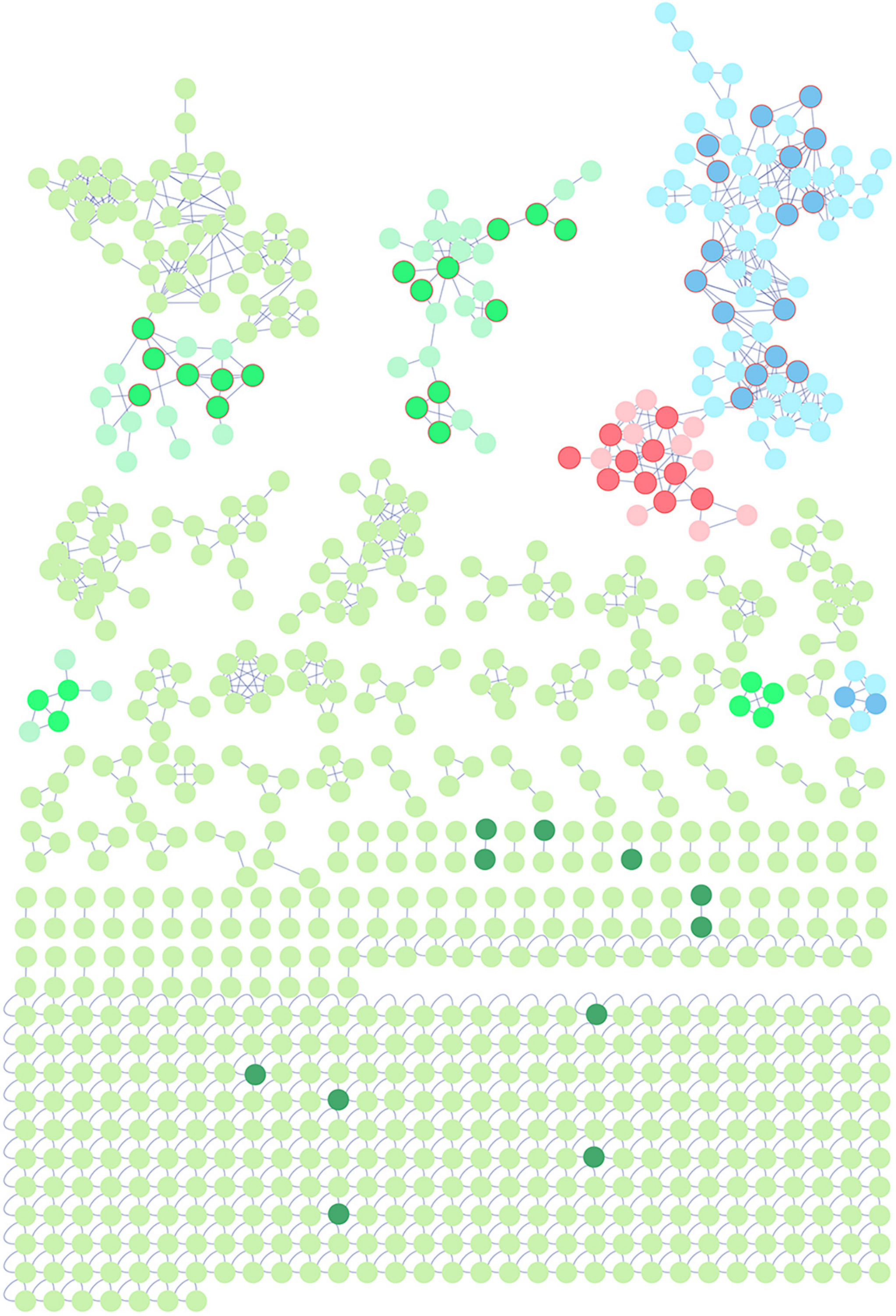

Figure S2. The molecular networking for LJF.

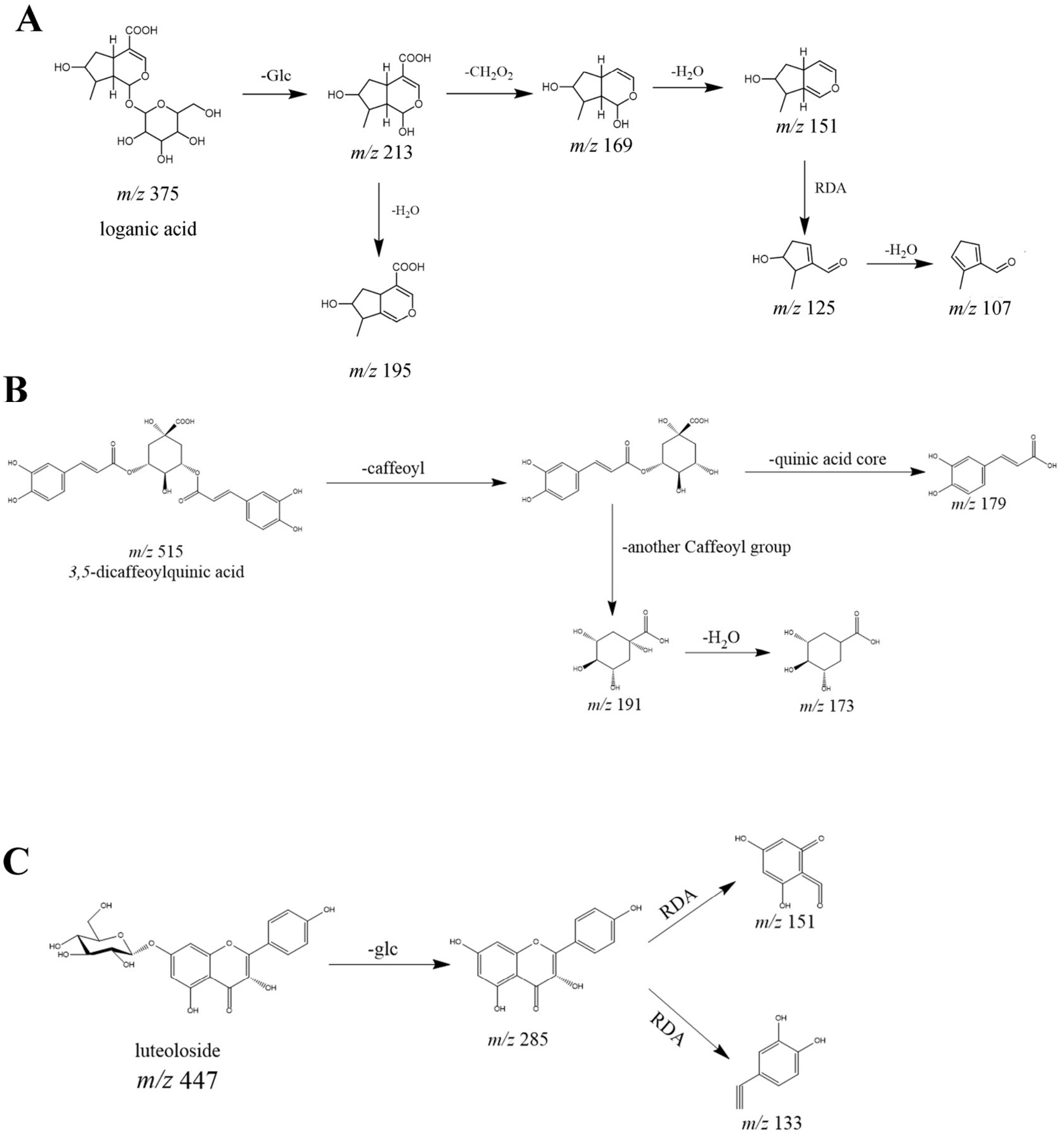

Figure S3. Proposed fragmentation pathways of representative iridoids (A) , phenolic acids (B) and flavonoids (C) based on MS/MS analysis.

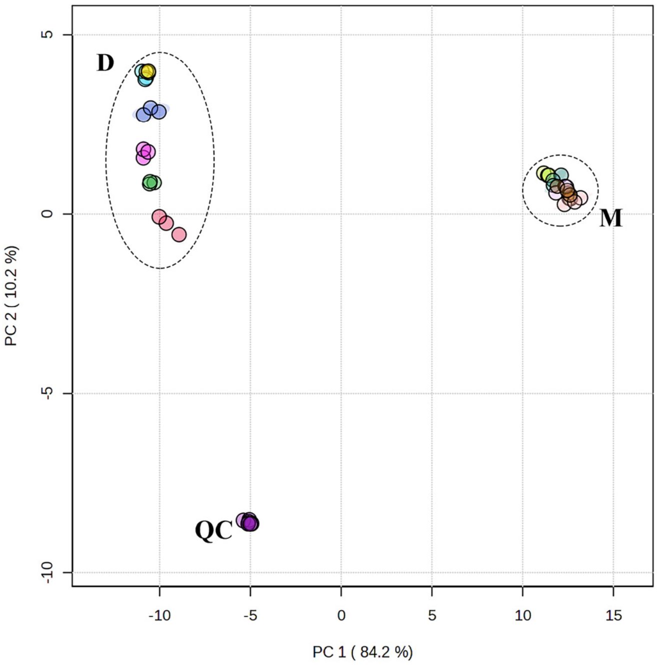

Figure S4. Score plot of PCA analysis among the QC samples, different decoction times (D) and different maceration times (M) samples.

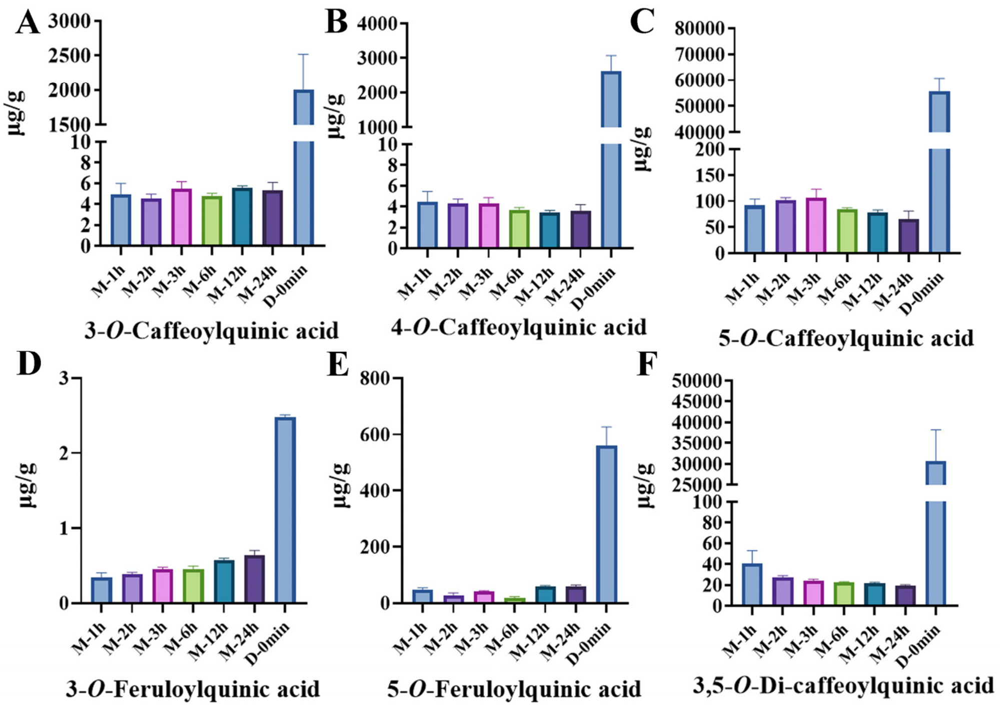

Figure S5. The content of phenolic acids during maceration process (1 h, 2 h, 3 h, 6 h, 12 h and 24 h) and decoction process (0 min).

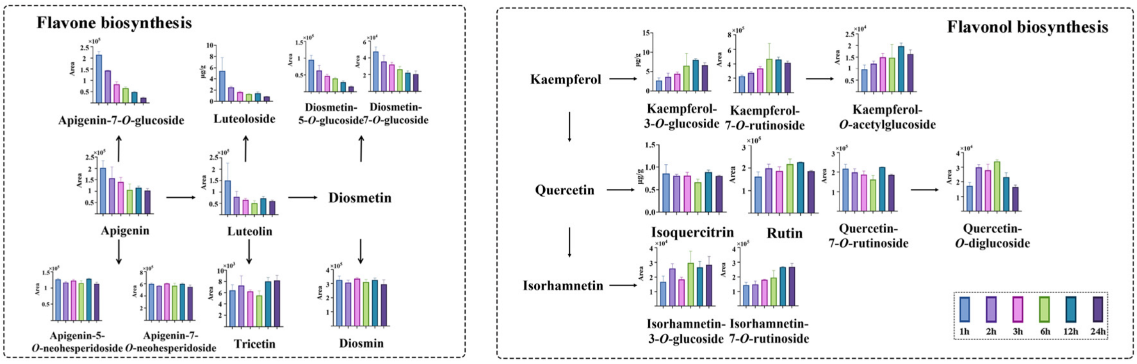

Figure S6. Distribution of flavonoids in the macerated samples.
